# Correction: An intercomparison of models predicting growth of Antarctic krill (*Euphausia superba*): The importance of recognizing model specificity

**DOI:** 10.1371/journal.pone.0333273

**Published:** 2025-09-24

**Authors:** Dominik Bahlburg, Sally E. Thorpe, Bettina Meyer, Uta Berger, Eugene J. Murphy

Fig 4 is incorrect and there are a number of errors in the caption. Please see the correct, complete Fig 4 caption here.

**Fig 4:**
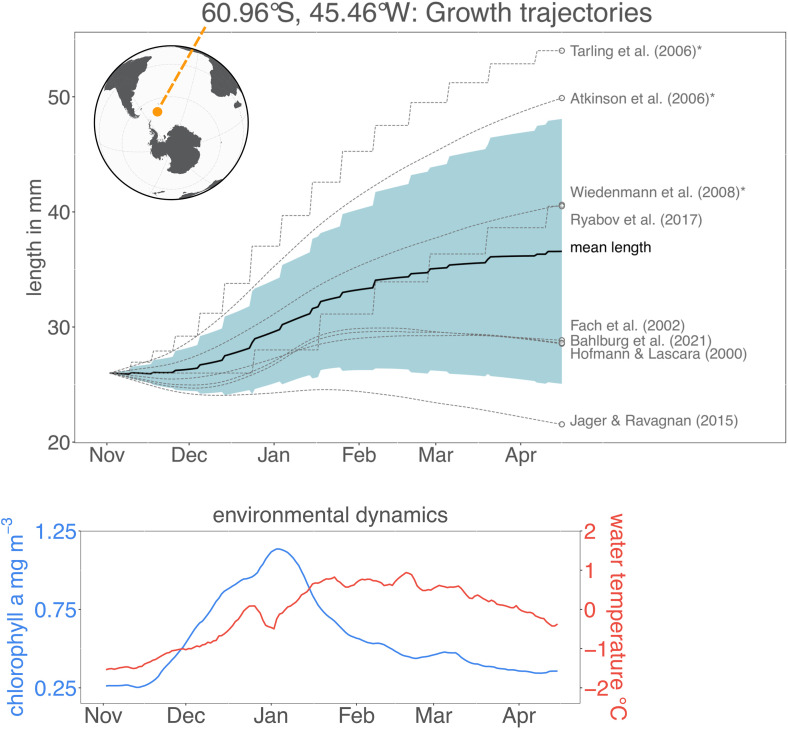
Top: Growth trajectories predicted by the different growth models for a location close to the South Orkney Islands—the exact location is shown in the map in the top left corner. The blue shaded area depicts the standard deviation of the outputs of all models at a given timepoint. The models strongly diverge with some models predicting very high (Tarling et al. (2006) [52]) and others predicting very low (Jager and Ravagnan (2015) [48]) growth. Bottom: Environmental data (sea surface temperature and chlorophyll a concentration) time series used to drive the growth trajectories in the top panel. The three empirical models are labelled with an asterisk. For body sizes <35 mm, the models of Atkinson et al. (2006) [44] and Tarling et al. (2006) [52] operate using the “juvenile” parameterizations for daily growth rate, growth increment and IMP, for sizes >35 mm the model of Atkinson et al. (2006) [44] uses the “all krill” parameterizations, the model of Tarling et al. (2006) [52] the “adult female” paramterization for growth increment and IMP.).
