# Supplementary figures and images for: An intercomparison of models predicting growth of Antarctic krill (Euphausia superba): The importance of recognizing model specificity
Source: PLoS One. 2023 Jul 28;18(7):e0286036. doi: 10.1371/journal.pone.0286036 (PMC10381086; doi:10.1371/journal.pone.0286036)

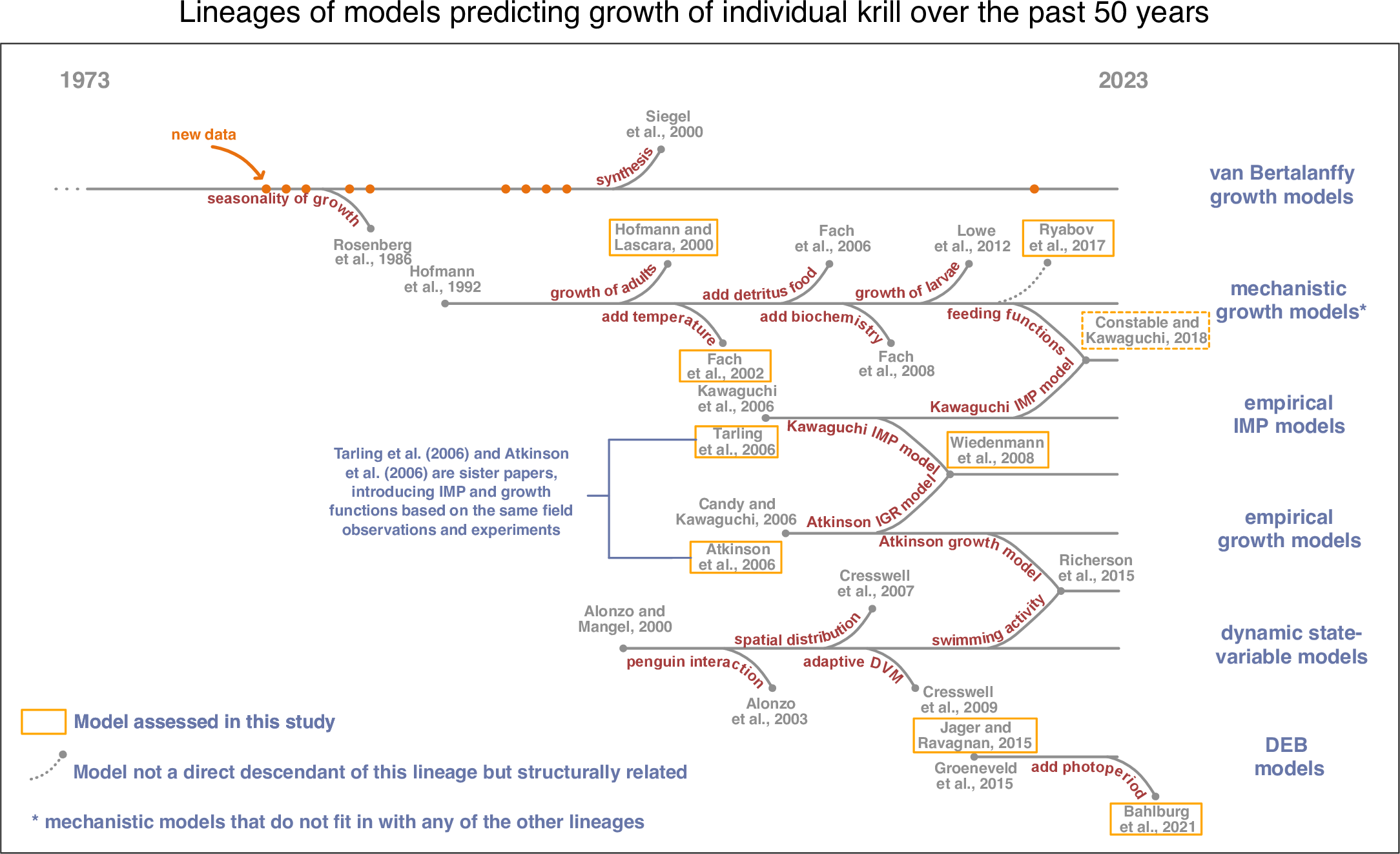

Supplement: S1 Fig — IMP—intermoult period, DEB—Dynamic Energy Budget, IGR—Instantaneous Growth Rate, DVM—Diel Vertical Migration. (TIF) [file pone.0286036.s001.tif]

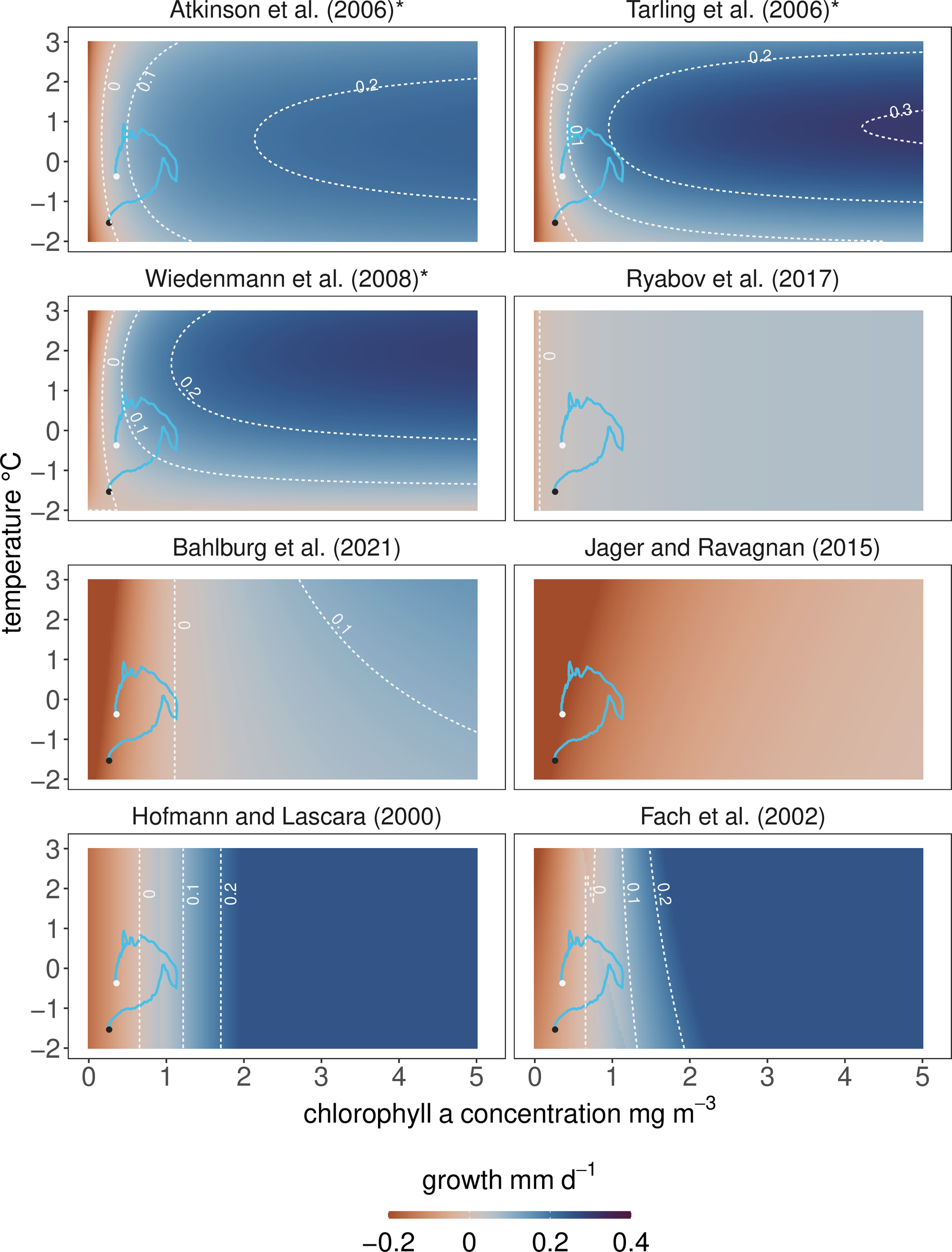

Supplement: S2 Fig — Blue lines as per Fig 5. (TIF) [file pone.0286036.s002.tif]

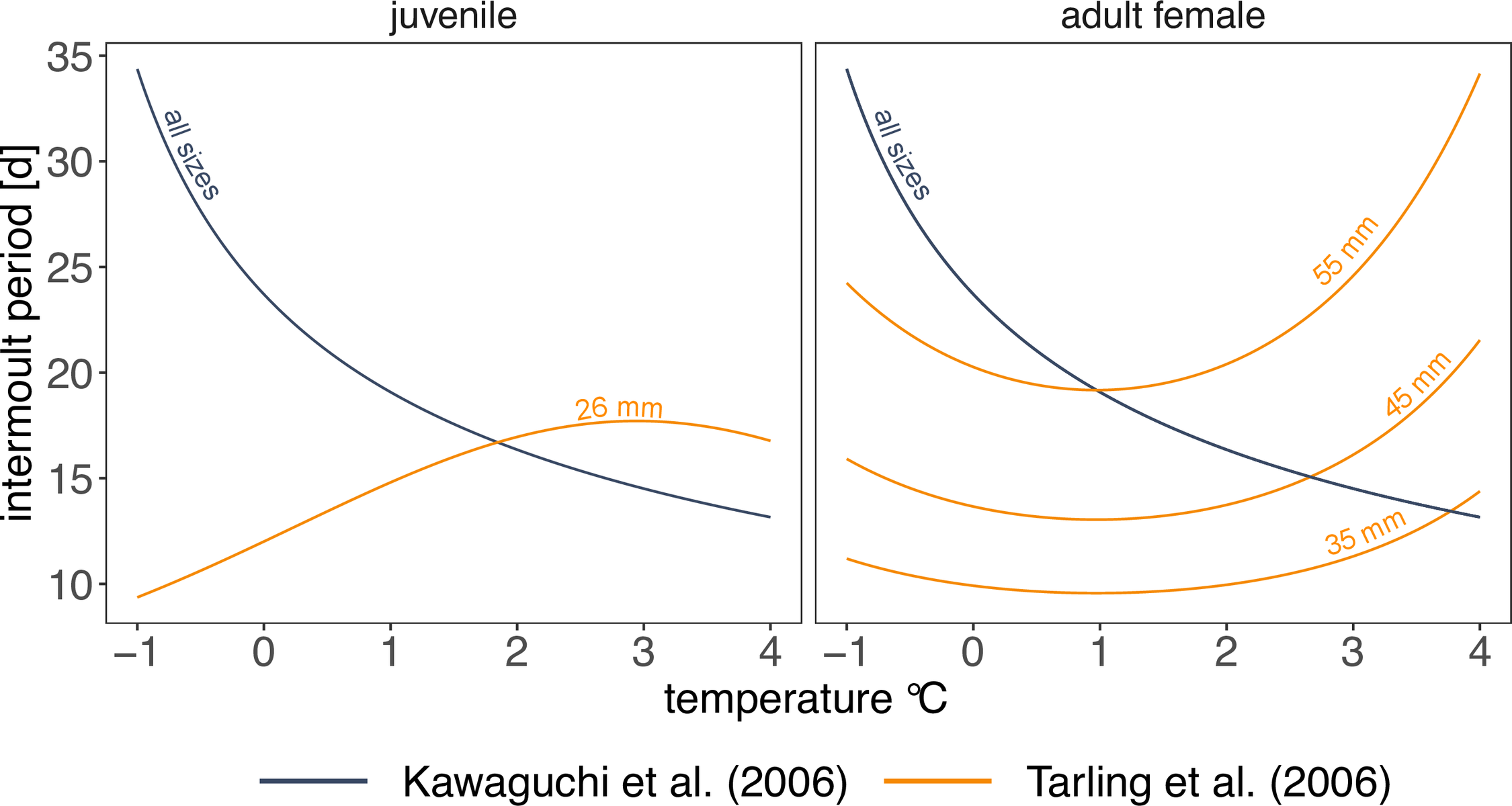

Supplement: S3 Fig — (TIF) [file pone.0286036.s003.tif]

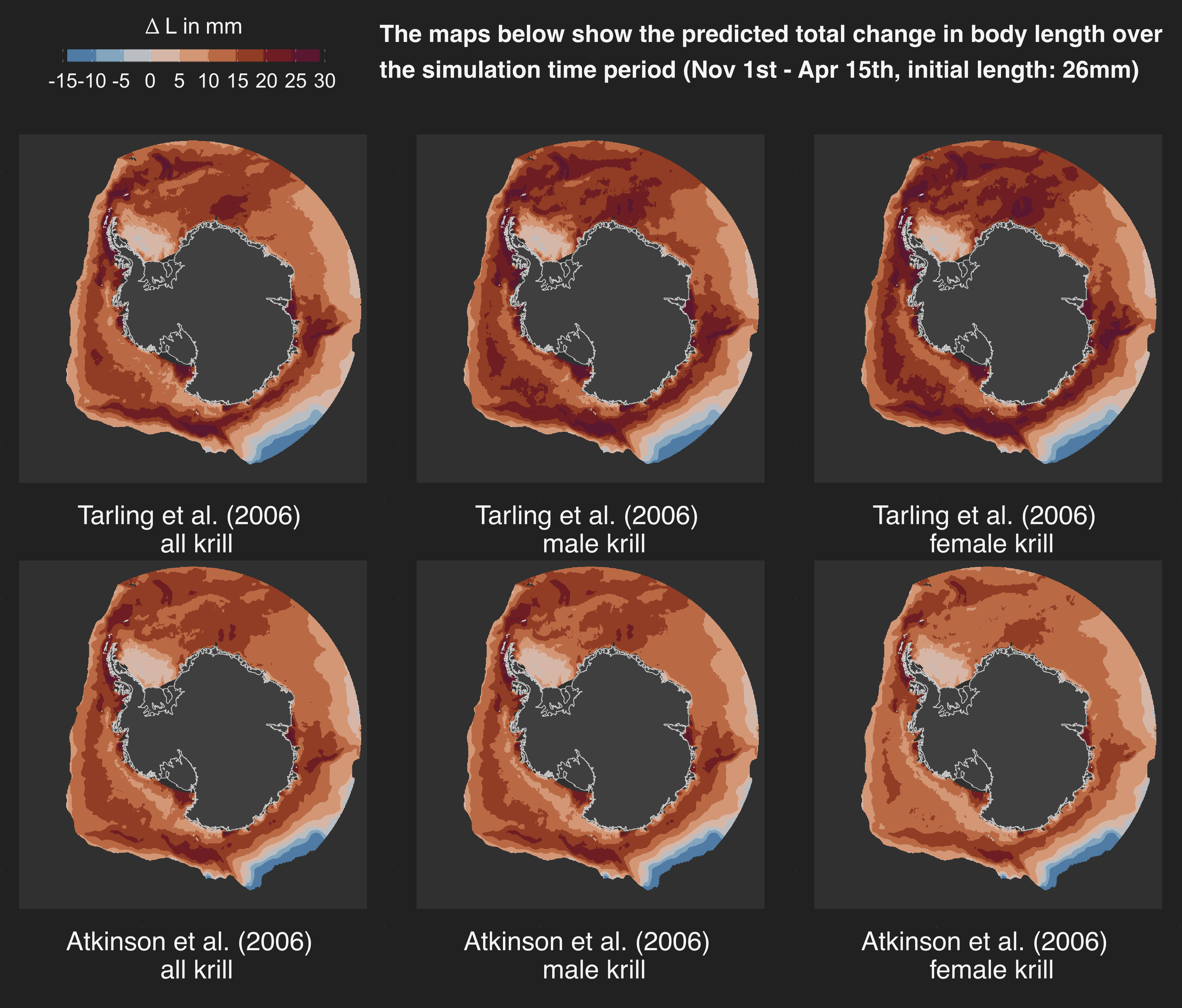

Supplement: S4 Fig — Note that for krill <35 mm, the models always operate in their “juvenile” parameterization. (TIF) [file pone.0286036.s004.tif]

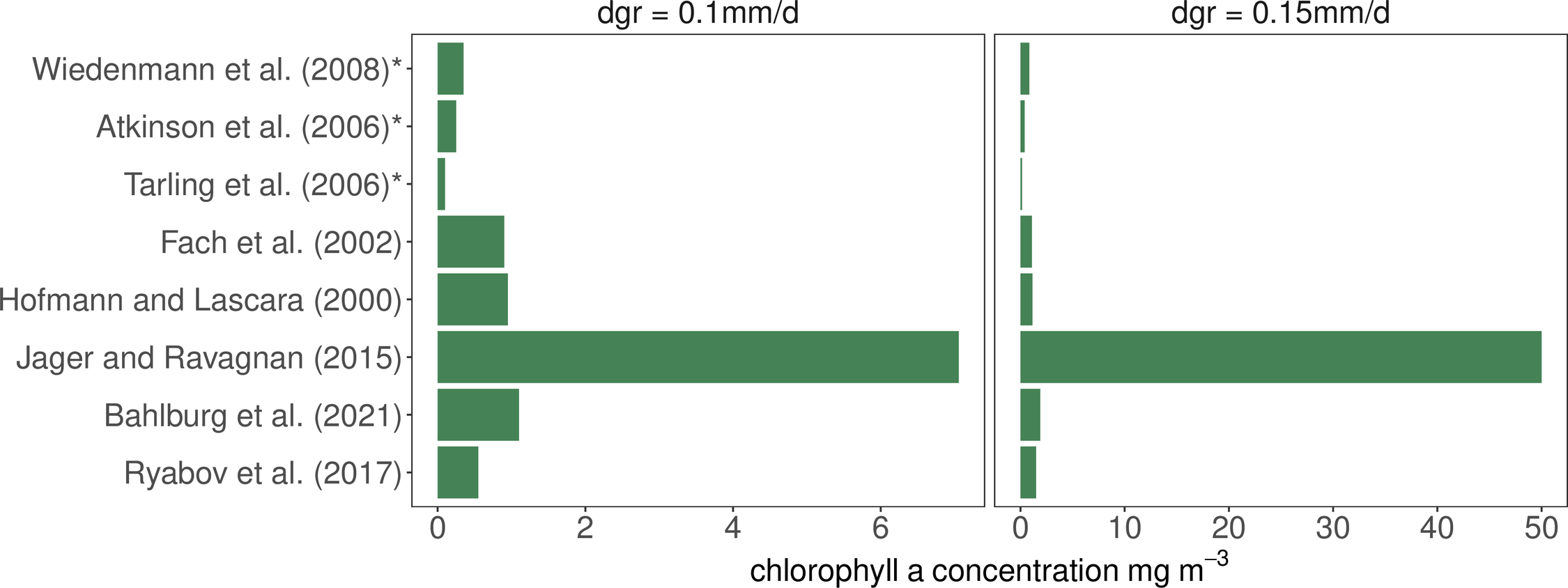

Supplement: S5 Fig — Empirical models are labelled with an asterisk. (TIF) [file pone.0286036.s005.tif]

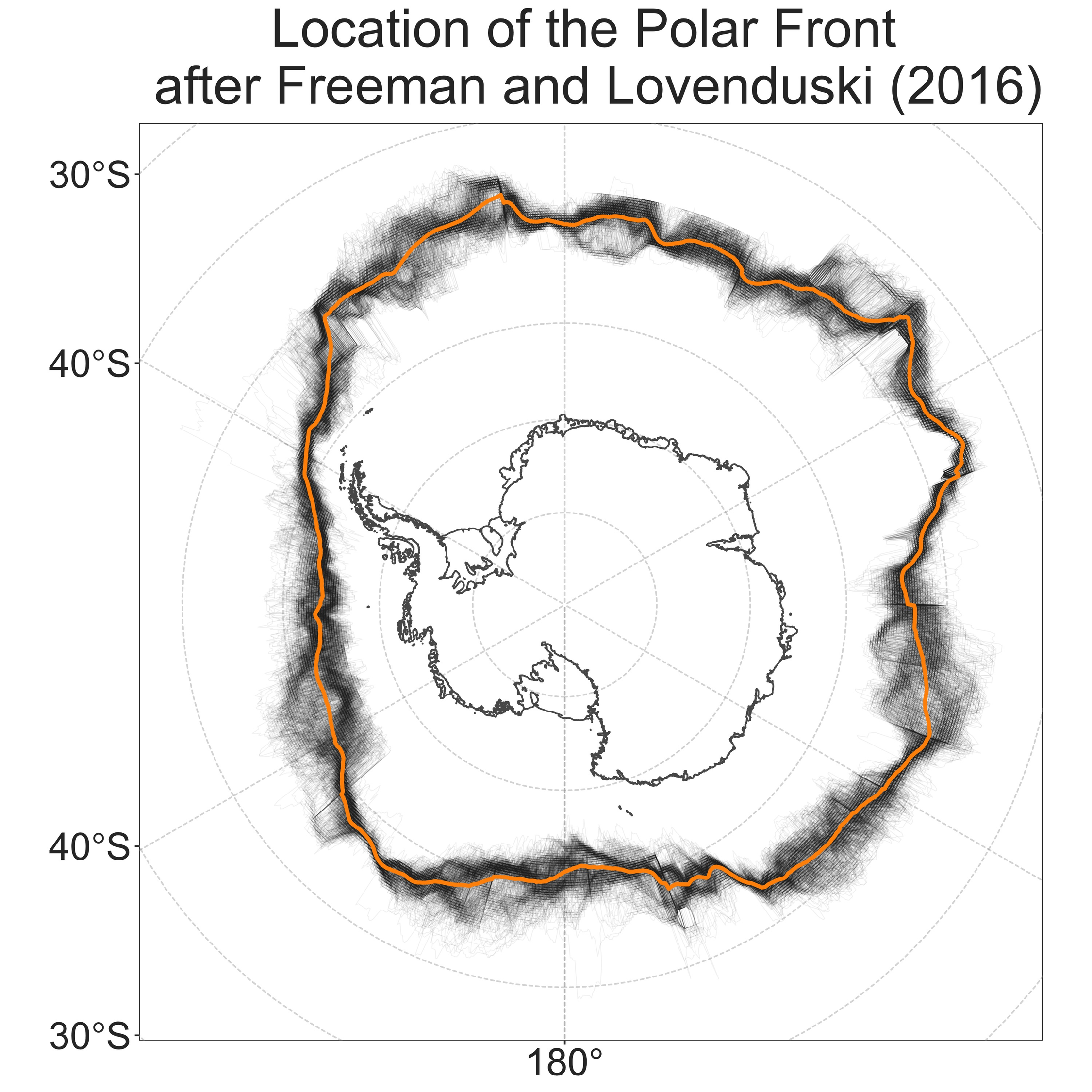

Supplement: S6 Fig — The black lines show the weekly locations of the Polar Front from 2002 to 2014, and the orange line the mean used to define the northern boundary of the model simulations. (TIF) [file pone.0286036.s006.tif]
