# Supplementary material for: An intercomparison of models predicting growth of Antarctic krill (Euphausia superba): The importance of recognizing model specificity
Source: PLoS One. 2023 Jul 28;18(7):e0286036. doi: 10.1371/journal.pone.0286036 (PMC10381086; doi:10.1371/journal.pone.0286036)
Supplement: Attachment — Submitted filename: BahlburgEtAlPlosOneS1_File_commented.pdf [file pone.0286036.s011.pdf]

Supplementary Information

Intercomparison of growth models for Antarctic krill (*Euphausia superba*): towards a generalised understanding

Dominik Bahlburg[1,2*], Sally Thorpe[3], Bettina Meyer[4,5,6], Uta Berger[1], Eugene Murphy[3],

1 Technische Universität Dresden, Dresden, Germany
2 Helmholtz Centre for Environmental Research, Leipzig, Germany
3 British Antarctic Survey, Cambridge, United Kingdom
4 Carl-von-Ossietzky Universität, Oldenburg, Germany
5 Alfred Wegener Institute for Polar and Marine Research, Bremerhaven, Germany
6 Helmholtz Institute for Functional Marine Biodiversity, Oldenburg, Germany

Additional Figures

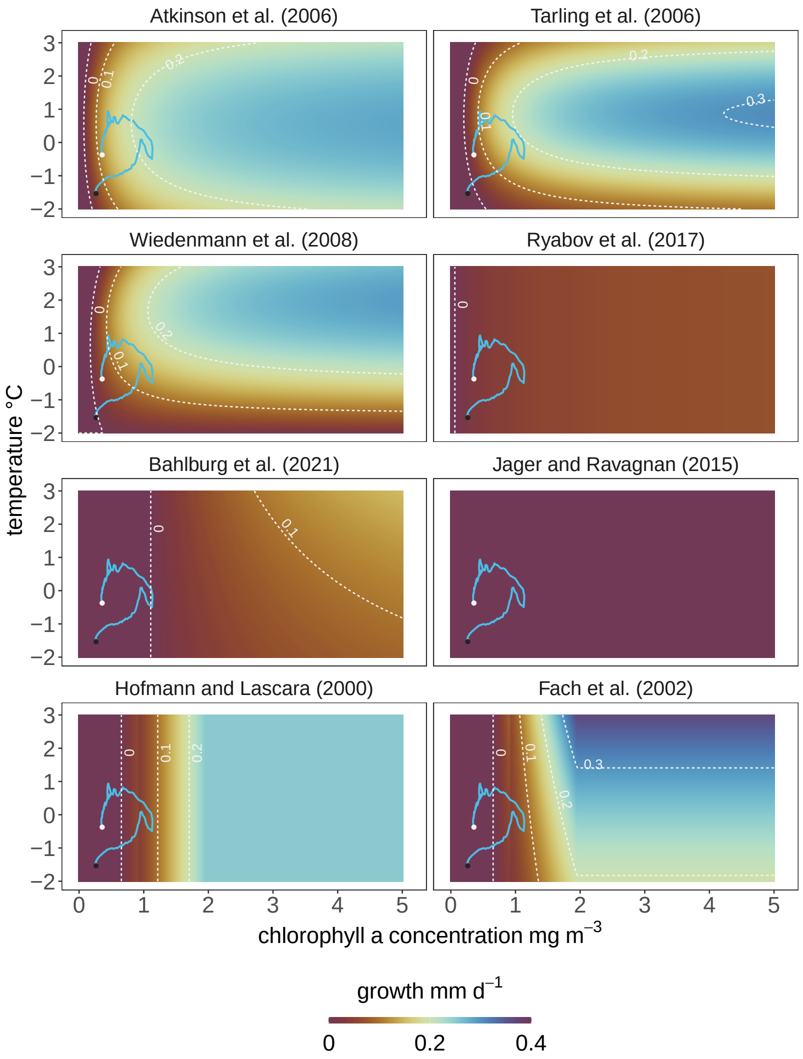

Atkinson et al. (2006)
Tarling et al. (2006)
Wiedenmann et al. (2008)
Ryabov et al. (2017)
Bahlburg et al. (2021)
Jager and Ravagnan (2015)
Hofmann and Lascara (2000)
Fach et al. (2002)
temperature °C
chlorophyll a concentration mg m^−3
growth mm d^−1
0
0.2
0.4

S1 Fig.

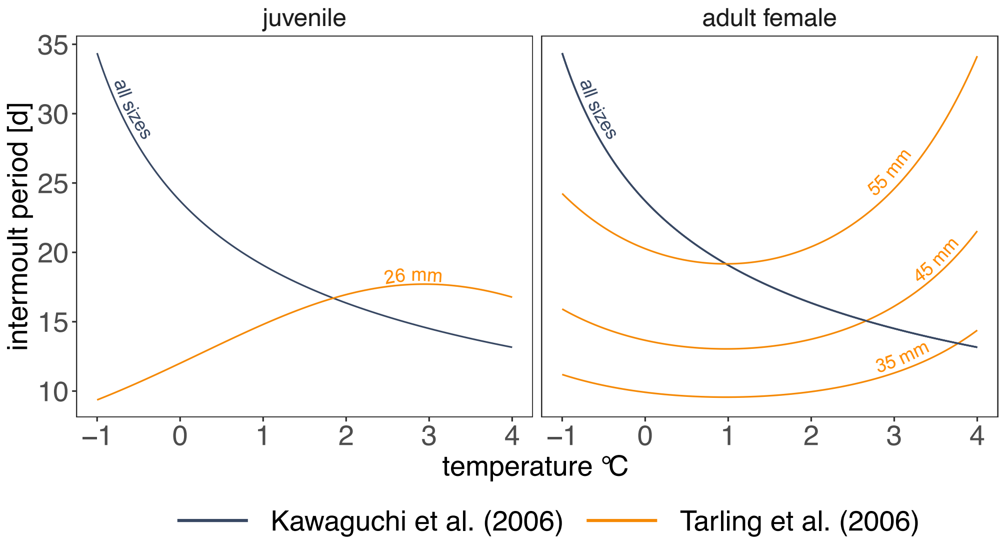

juvenile
adult female
intermoult period [d]
35
30
25
20
15
10
all sizes
26 mm
55 mm
45 mm
35 mm
–1
0
1
2
3
4
temperature ℃
Kawaguchi et al. (2006)
Tarling et al. (2006)

S2 Fig.

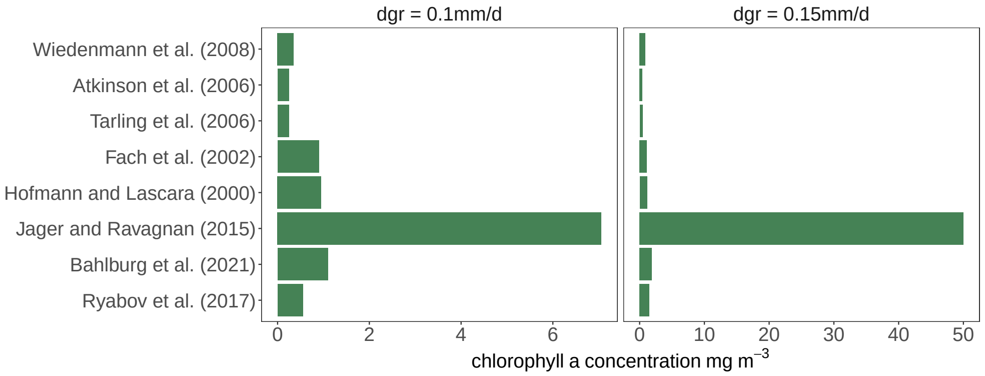

dgr = 0.1mm/d
dgr = 0.15mm/d
Wiedenmann et al. (2008)
Atkinson et al. (2006)
Tarling et al. (2006)
Fach et al. (2002)
Hofmann and Lascara (2000)
Jager and Ravagnan (2015)
Bahlburg et al. (2021)
Ryabov et al. (2017)
0
2
4
6
0
10
20
30
40
50
chlorophyll a concentration mg m^{-3}

S3 Fig.

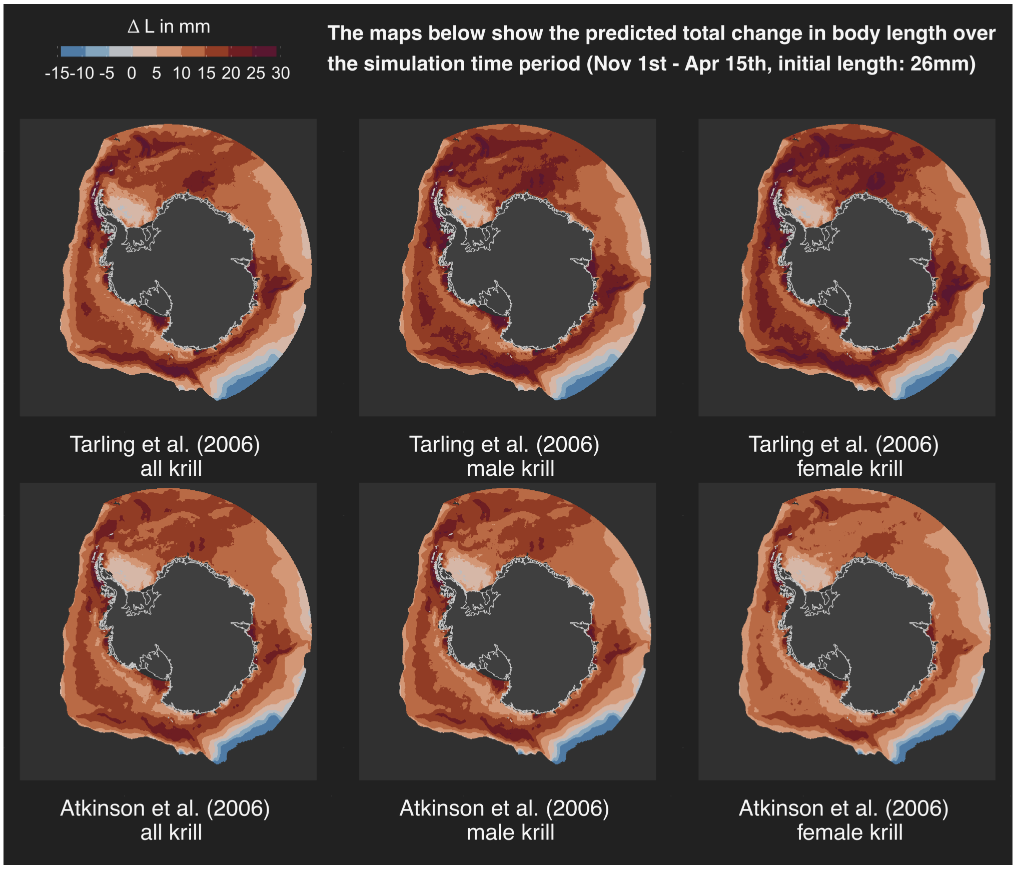

Δ L in mm
-15 -10 -5 0 5 10 15 20 25 30
The maps below show the predicted total change in body length over the simulation time period (Nov 1st - Apr 15th, initial length: 26mm)
Tarling et al. (2006) all krill
Tarling et al. (2006) male krill
Tarling et al. (2006) female krill
Atkinson et al. (2006) all krill
Atkinson et al. (2006) male krill
Atkinson et al. (2006) female krill

S4 Fig.

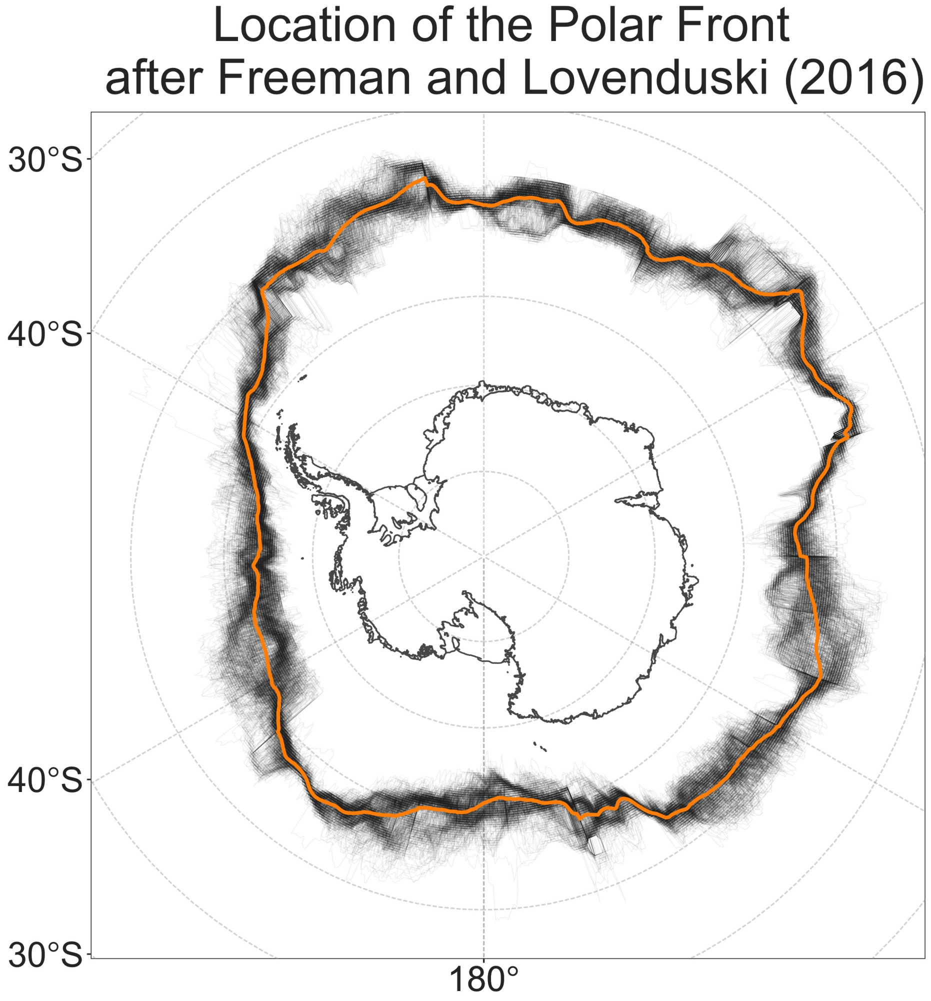
Location of the Polar Front
after Freeman and Lovenduski (2016)
30°S
40°S
40°S
30°S
180°

S5 Fig.

S1 File: Quality Control of the different model implementations

In this part, we validate our krill growth model implementations for correctness. This is done by running the different models using the environmental input data used in their original publication. We then compare the predicted growth trajectories with those that were originally reported. Original data (environmental data and growth trajectories) were extracted from the original manuscripts using a data extraction tool from ImageJ.

Hofmann and Lascara (2000)

In the quality control of our implementation, we simulated the growth trajectories of 3 individuals with starting lengths of 2, 22 and 45mm over 468 days as was done in the original paper. The simulation was performed twice, once excluding ice algae as a food source (trajectories shown in Figure 7 of the paper) and including ice algae (Figure 8 of the paper).

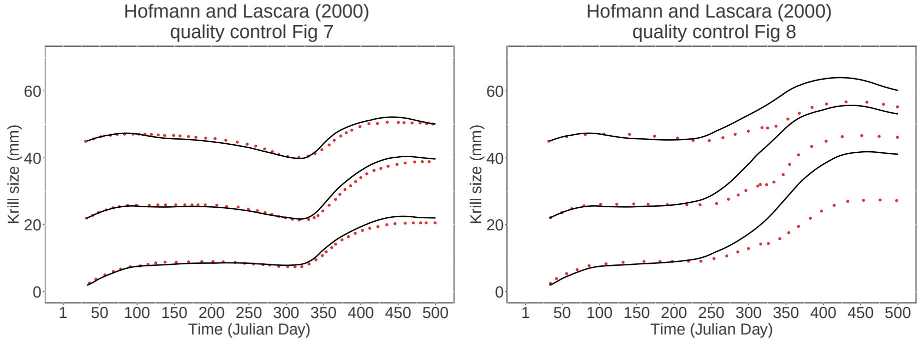

Figure 1 Comparing growth trajectories of our implementation of the model of Hofmann and Lascara (2000, black lines) and the original model (red points, left: without ice algae, right: with ice algae).

The model simulations slightly diverge from the original trajectories but general patterns are reasonably well reproduced - possible explanations for the divergences could come from differences in the numerical integration, inaccuracies of our environmental time series which was extracted from Figure 6 or minor differences in parameter values that are reported in the original manuscript and the Fortran-files of the model that Eileen Hofmann provided us with. The Fortran-files include minor modifications of the model that were not described in the manuscript. We did not include these modifications as they actually worsen the fit (modifications are that daily rations are capped at 20% and set to 0 below 1.5%)

Including ice algae as a second food source results in stronger differences between our implementation and the originally reported trajectories. Growth is strongly overestimated in our version. We went through the Fortran files and could not really find the problem yet. Unfortunately, we do not have access to the original model input files which are being used in the Fortran-script to parameterize ingestion and assimilation of sea ice biota. We also could not find any mistakes in our implementation.

In our project, we restrict the simulations to summer growth and excluded ice algae as a food source - also because no circumpolar predictions of sea ice biota (accessible for krill) exist. Therefore, potential inaccuracies in the circumpolar model simulations are comparable with those shown in the left panel of Figure 1.

Conclusion: We conclude that our implementation reasonably well reproduces growth trajectories that the original model would predict. For future studies, it would

be good to identify the differences between our model version and the original model, especially regarding the inclusion of sea ice biota as a food source.

Ryabov et al. (2017)

The growth component of the Ryabov et al. (2017) model was directly provided by Alexey Ryabov. He shared the growth component of his model using an environmental data time series that we previously provided him with. His model version was implemented in Matlab files that we translated into R.

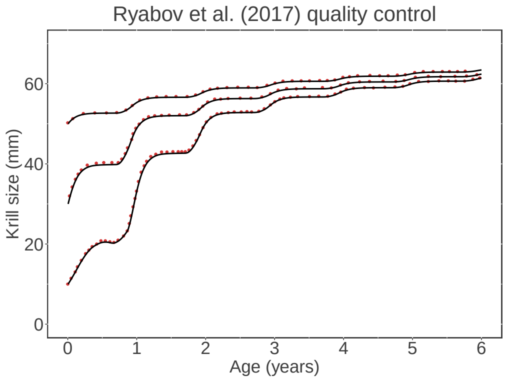

Figure 2 Comparing growth trajectories of our implementation of the model of Ryabov et al. (2017, black line) and the original model (red points).

Conclusion: The model is correctly implemented and can be used as it is.

Fach et al. (2002)

The model of Fach et al. (2002) is a modification of Hofmann and Lascara (2000). It includes heterotrophic food as a third food source as well as temperature-dependency of metabolic costs. There are many figures in the original manuscript showing different time series of environmental factors. At this point, we are not sure which timeseries are combined in the different simulations. As the modifications made to the original model are rather minor (Fach et al. (2002) has a temperature-dependence of metabolism added, otherwise both models are identical), we assume that the quality assessment yields similar results as the one on Hofmann and Lascara (2000).

Atkinson et al. (2006):

In Atkinson et al. (2006), no growth trajectories are shown but predicted daily growth rates for individuals of three different length at two temperatures over a chlorophyll a gradient are presented. We compare the predictions of our implementations (black lines) with those shown in the Figure 6 in the manuscript (red points).

The predicted growth rates from our implementation perfectly match those reported in the paper.

Conclusion: The model is correctly implemented and can be used as it is.

Tarling et al. (2006)

The original manuscript does not provide growth trajectories. However, it provides model estimates for intermoult periods for different stages, environmental conditions and body lengths in Figure 7 and 8 of the original manuscript.

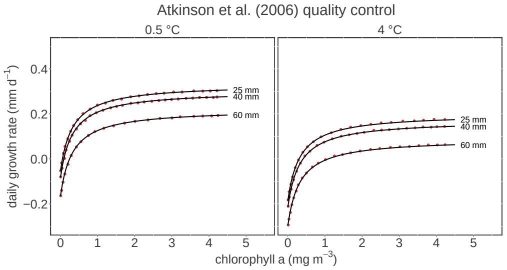

Figure 3 Comparing daily growth rates of our implementation of the model (black lines) of Atkinson et al. (2006) and the original model (red points) as presented in Figure 6 of the original paper.

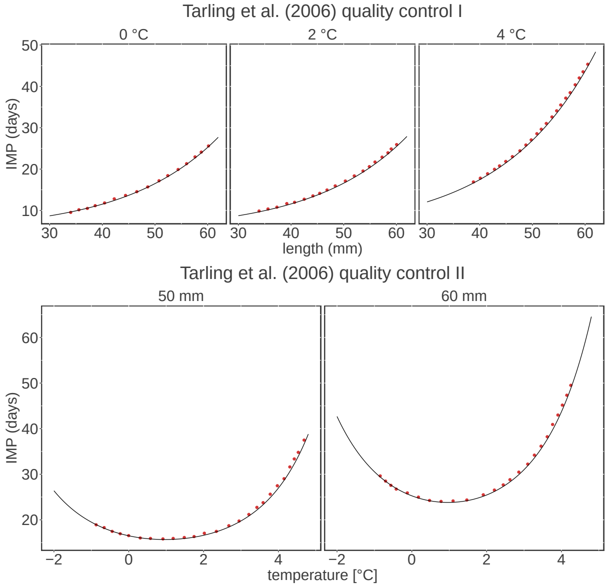

Figure 4 Comparing IMPs of our implementation of the model of Tarling et al. (2006, black lines) and the original model (red points). Top panel: Comparison of the data reported in Figure 7 in of the original manuscript, bottom panel: Figure 8 of the original manuscript

Conclusion: The results shown in Tarling et al. (2006) only allow for validating the intermoult period-function. Based on the previous figures, we conclude that this component is correctly implemented. The growth-increment function used in the simulations (from Atkinson et al. (2006)) was not visualized in its original publication, but given the relative simplicity of this function, it is unlikely that we have implemented it incorrectly.

Add-on: We note that the model of Tarling et al. (2006) produces growth trajectories that differ from those predicted by the model of Atkinson et al. (2006). In the following, we take a more detailed look at the mechanisms causing these differences.

It can be seen that the growth increment function of the model of Atkinson et al. (2006) for juvenile krill has a similar shape than the daily growth rate-function for different temperatures and chlorophyll a concentrations (Figure 5) with maximum growth predicted for temperatures around 1 °C and chlorophyll a concentrations >1.5 mg mm^{-3}. However, the IMP-function of the model of Tarling et al. (2006) predicts minimum intermoult periods for the lowest temperatures (-1 °C) which corresponds to a high moulting frequency. Since the intermoult periods are an important driver of

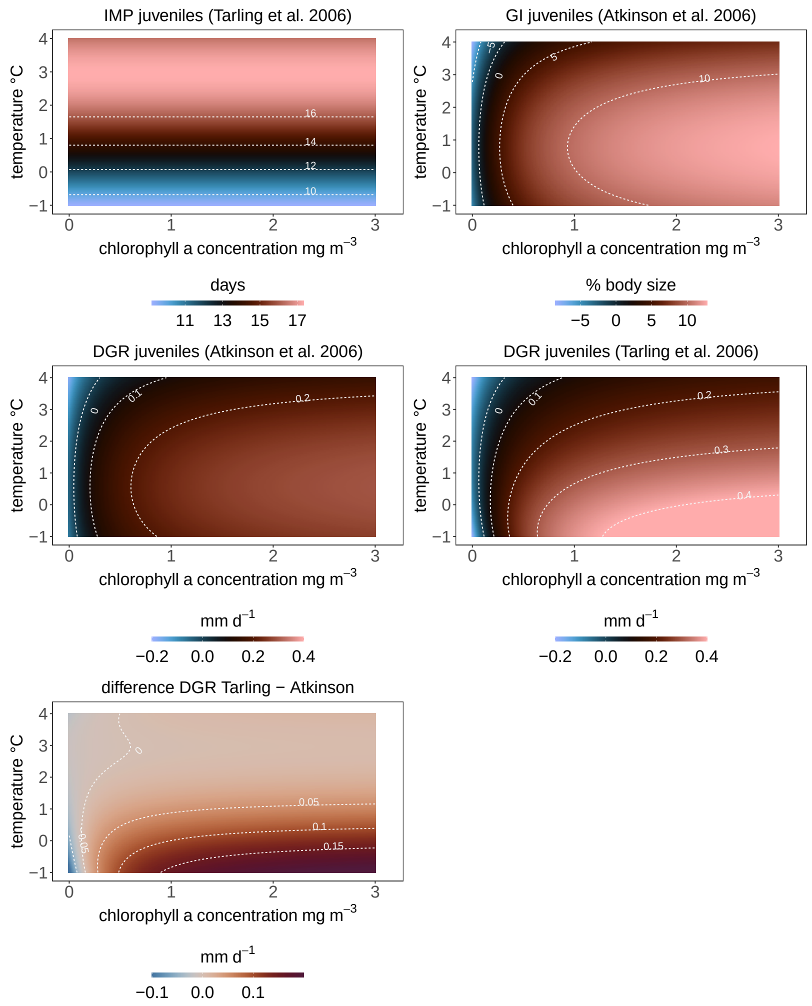

Figure 5 Diagnostic plots for investigating drivers of the differences between the models of Atkinson et al. (2006) and Tarling et al. (2006, intermoult period- and growth increment function). Plots are based on a 26mm krill individual and the ”juvenile” parameteriations of both models. IMP - intermoult period, GI - growth increment. DGR - daily growth rate

growth in the model of Tarling et al. (2006), the predicted daily growth rates also peak at the lowest temperatures.

For completeness, we also compared the growth properties predicted by both models for adult krill.

For adult female krill, we see that the intermoult period function of the model of Tarling et al. (2006) has a different shape to that for juvenile krill. Minimum intermoult periods, and therefore most frequent moultings, are predicted for temperatures around 1 °C (Figure 6). Generally, these intermoult periods are longer than the ones predicted for juvenile krill which results in reduced growth. In addition, the growth increment function from Atkinson et al. (2006) predicts comparatively less growth (¡10% growth increment for all environmental conditions investigated here). Since both the intermoult period function and the growth increment functions take on values corresponding to high growth for temperatures of ∼1 °C, the daily growth rates predicted for adult females by the Tarling et al. (2006) model peak around the same temperatures. This is

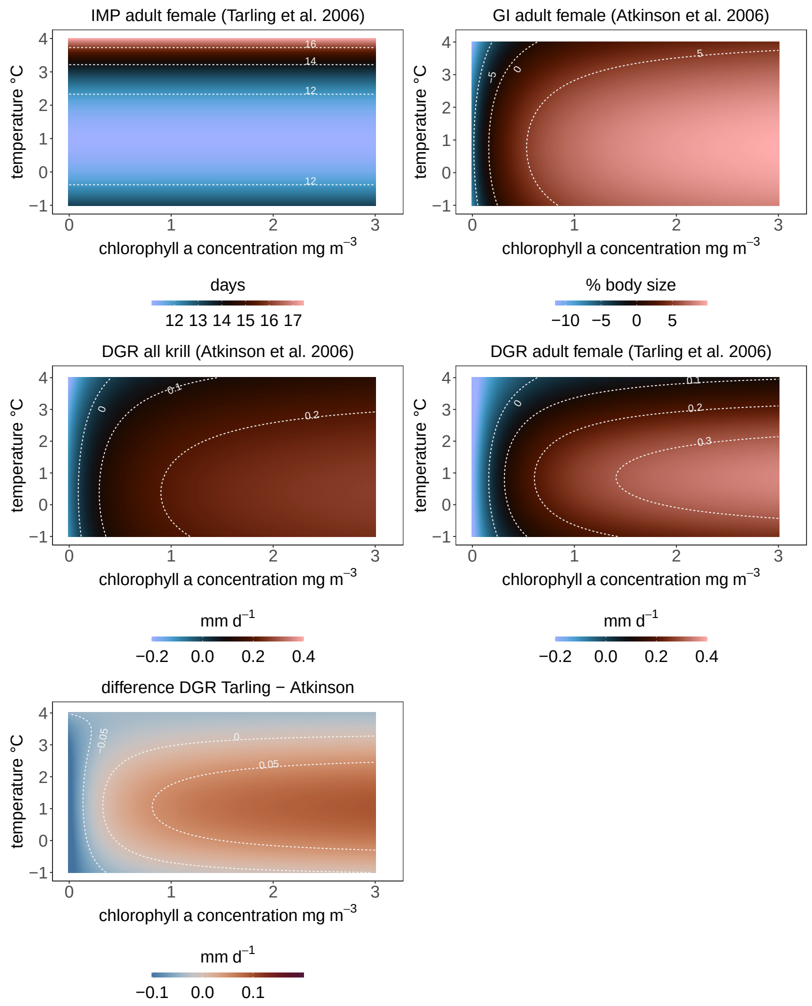

Figure 6 Diagnostic plots for investigating drivers of the differences between the models of Atkinson et al. (2006) and Tarling et al. (2006). Plots are based on a 40mm krill individual and the "adult female" parameteriations for the model of Tarling et al. (2006, intermoult period- and growth increment function) and the "all krill" parameterization for the model of Atkinson et al. (2006). IMP - intermoult period, GI - growth increment. DGR - daily growth rate

in contrast to the juvenile parameterisation, where growth peaks at the lowest temperatures (-1 °C). The differences in daily growth rates predicted by both models are slightly lower compared to juveniles when using the adult female-version the model of Tarling et al. (2006) and the all krill model of Atkinson et al. (2006). We also compared the female and the male-versions of both models which resulted in increased differences compared to the adult female version of Tarling et al. (2006) vs. all krill version of Atkinson et al. (2006).

Wiedenmann et al. (2008)

The model structure of Wiedenmann et al. (2008) is very similar to Tarling et al. (2006) but instead of using the intermoult period model from Tarling et al. (2006), it uses the intermoult period-model presented in Kawaguchi (2006), in which intermoult period is a function of water temperature only. Other than that, both models are identically implemented using the Atkinson et al. (2006) all krill-growth increment function for predicting growth at the time of moulting. We can compare the intermoult period model used in our implementation of Wiedenmann et al. (2008) with the results shown in Figure 5 in Kawaguchi et al. (2006).

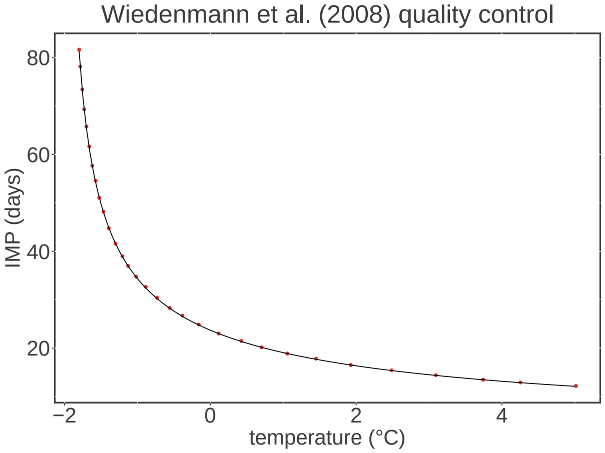

Figure 7 Comparing intermoult periods of our implementation of the model of Kawaguchi et al. (2006, black line) which is used in Wiedenmann et al. (2008) and the original model (red points). Data shown here come from Figure 5 in Kawaguchi et al. (2006)

Conclusion: Based on the same analyses done for Tarling et al. (2006), we assume that the model is correctly implemented.

Bahlburg et al. (2021)

This model was originally developed and implemented by the corresponding author and is publicly documented under https://github.com/dbahlburg/SERBIK. A minor change from the original model was made in our simulations by removing the "foodConversion"-constant of 0.8 which is multiplied with the ingested carbon. This was done since we realized that this constant is not included in the baseline model of Jager and Ravagnan (2015).

Jager and Ravagnan (2015):

This model assumed unlimited food in its original publication. When using the same assumption and the exact parameterization provided in Table 2 of the manuscript, our implementation systematically underestimates the originally reported daily growth rates as a function of body length at 0°C and 5°C:

Increasing the area-specific carbon assimilation constant slightly from 0.044 to 0.045 gives a better match. For our analyses, we worked with this ajusted parameter value.

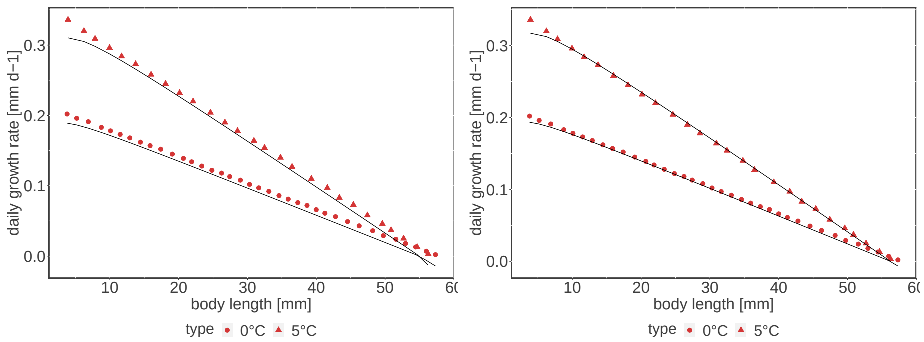

Figure 8 Comparing IMPs of our implementation of the model of Jager and Ravagnan (2015) with originally reported results. Left panel: Original results (red points) and reproduced results (black lines) at 0°C and 5°C using a area-specific carbon assimilation constant of 0.044, right panel: same simulations but using a area-specific carbon assimilation constant of 0.045

Constable and Kawaguchi (2018) - excluded from the main analysis

The model of Constable and Kawaguchi integrates parts of Hofmann and Lascara (2000) into an intermoult period framework. We compare our implementation with the results shown in Figure 4 and 5 in the original paper. It can be seen that in our implementation, growth rates are much higher than in the reported results in the paper. Dry mass is almost 3 times higher, reproduction occurs much more frequently and length increases at substantially higher rates.

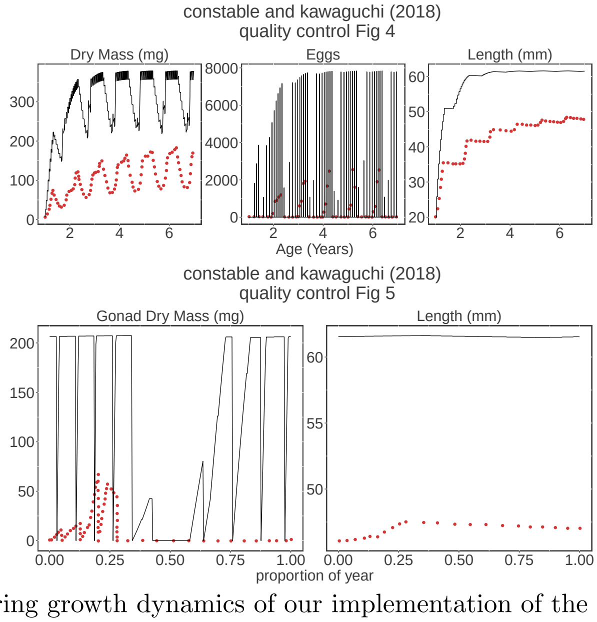

Figure 9 Comparing growth dynamics of our implementation of the model of Constable and Kawaguchi (2018). Top panel: Original results (red points) and reproduced results (black lines) using the environmental time series presented in the original manuscript and Figure 4 in the original paper, bottom panel: zooming into the dynamics of the gonad dry mass and body length (Figure 5 in the original paper)

A more detailed comparison of the energy dynamics in year 5 shows that gonad dry

mass is peaking at the threshold value in our simulation while it remains much lower in the original results. Similarly, the krill is considerably smaller (~45 mm vs. 65 mm) in the original results. It seems that in our implementation, the krill has much more energy available for growth and reproduction compared to the original simulations. This means that differences must exist in either the mechanisms of food assimilation or respiration. However, the underlying intermoult period-philosophy of the model is merely reflected in the dynamics shown in the original Figure 4 and 5 (we would expect a step-wise growth pattern similar to Tarling et al. 2006 or Wiedenmann et al. 2008). Krill growth seems to be continuous, energy dynamics look smooth (although there should be a sudden loss of body mass at the time of moulting due to the discard of the old carapace) and gonad dry mass does not show the dynamics we would expect based on the model mechanisms (gonad dry mass should be reduced to zero after each spawning, however there is only a partial reduction). Apparently, a considerable energy surplus leads to exaggerated growth and reproduction in our model. We took another look at the environmental time series presented in Figure 3 of the paper. According to the authors, it shows the dynamics of POC, temperature and day length used to create the model results shown in Figure 5 and 6 of the original paper. After taking a closer look, we realized that the POC concentrations in this time series are very high. Approximating the concentrations as chlorophyll a concentrations reveals that the krill individual is exposed to concentrations of >5 mg Chla m^{-3} in at least 6 months (using a conversion constant of ChlA:POC = 50 as in Hofmann and Lascara (2000)).

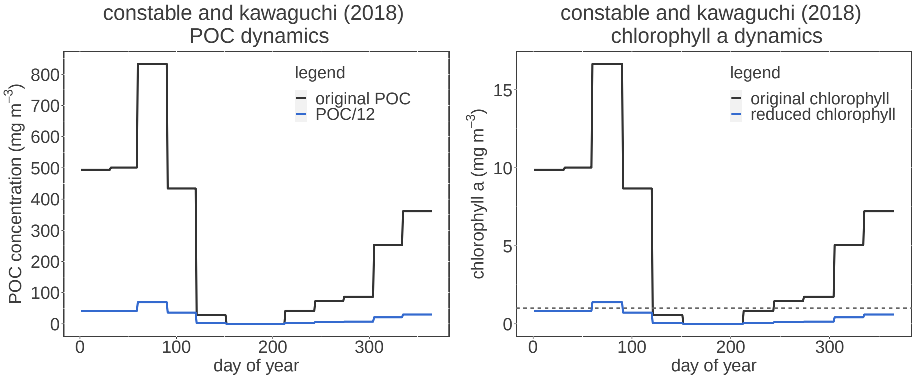

Figure 10 Environmental time series as presented in Figure 3 in Constable and Kawaguchi (2018). Left panel: POC dynamics - original data as black line, downscaled data as blue line, right panel: chlorophyll a dynamics - original data as black line, downscaled data as blue line

These very high food concentrations could explain some of the differences between our model and the trajectories from the original paper. Arguably, the displayed environmental time series deviates from the actual one used for model simulations. To see how our implementation performs using more realistic food concentration, we reduced the POC concentrations by an arbitrary factor of 12 (blue line, the dotted line marks the 1 mg m^{-3} threshold). This way, approximated chlorophyll a concentrations vary between 0 and 1.4 mg m^{-3} with an annual average of ~0.5 mg m^{-3}. The modified time series might be on the lower end of field chlorophyll a dynamics but still representative of the Southern Ocean environment. We re-ran the simulations with the reduced food concentrations and obtained the following results:

In the more detailed analysis, we see that our model typically misses the first two annual spawning events shown in the original results. The number of released eggs in following spawnings are slightly higher in our model (indicated by the higher gonad dry mass). There are strong indications that the originally presented environmental time

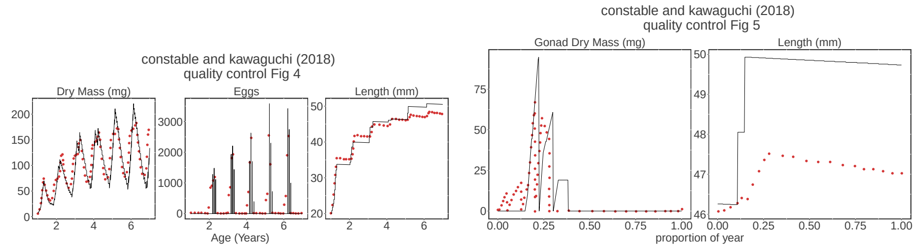

Figure 11 Comparing growth dynamics of our implementation of the model of Constable and Kawaguchi (2018) but this time using downscaled food concentrations. Top panel: Original results (red points) and reproduced results (black lines) using the environmental time series presented in the original manuscript and Figure 4 in the original paper, bottom panel: zooming into the dynamics of the gonad dry mass and body length (Figure 5 in the original paper)

series is not the one used for doing the actual simulations. Downscaling food concentration with a factor of 12x leads to results that are much closer to the original ones, although not identical. This leaves the following options:

1. Our model implementation is wrong - but this implies that the correct model needs unrealistically high food concentrations to predict realistic krill development

2. The authors used a different environmental time series, not only with reduced chlorophyll a concentrations but also differing dynamics of day length and temperature - this would explain why our results are similar but not identical after downscaling food concentrations

3. A mix of the above - our models differ in some details leading to slightly different predictions and a different environmental time series was used

Our implementation likely represents a close but not identical version of the original growth model. Since we cannot quantify the scale of agreement, we excluded it from the main analysis. However, the model code is available in the code repository.
