# Supplementary material for: An intercomparison of models predicting growth of Antarctic krill (Euphausia superba): The importance of recognizing model specificity
Source: PLoS One. 2023 Jul 28;18(7):e0286036. doi: 10.1371/journal.pone.0286036 (PMC10381086; doi:10.1371/journal.pone.0286036)
Supplement: Attachment — Submitted filename: Reviewer-comments.docx [file pone.0286036.s009.docx]

This paper compares an ensemble of empirical and mechanistic krill growth models, and assesses them based on their generality – i.e. their suitability for extrapolation in space and time. The authors conclude that the model results are sensitive to the data to which the model was calibrated, and advocate for these strengths and limitations to be accounted for in future studies applying these models, as well as the value of model re-calibration and code transparency to allow for reproducibility.

I see this study as a very valuable addition to the field of krill habitat modelling and thank the authors for their work. Firstly, krill growth models are often used to predict the impacts of climate change on krill habitat (e.g. Hill et al. 2013, Veytia et al. 2020, Sylvester et al. 2021) and an inter-comparison of growth models is valuable for assessing strengths and biases within these applications. Secondly, the availability of these models within open-source code is a valuable resource in and of itself that will assist in future reproducibility. Lastly, the authors have highlighted knowledge gaps that are important for reducing biases in large-scale and future assessments of krill habitat.

Overall, the paper and its accompanying code are well-structured and well written. I have made comments and added questions throughout the main text and supplementary files to improve clarity and flow of argument as well as to provide additional rationale/discussion. I therefore recommend publication with these moderate adjustments (see comments in attached files).

My main comments are provided below:

- Introduction
  - When using the word “generality” in terms of the analysis aims, I think this needs an extra sentence or two to provide a more explicit definition. The way “generality” appears to be used here refers to how suitable a model is for extrapolation in geographic space, in time, and in environmental space (i.e. outside the environmental conditions it was calibrated for). By extrapolating models across these different dimensions, it is possible to validate the model predictions with existing knowledge on krill distribution and habitat quality. This helps define the limits of how a model may be suitably applied for of current and future habitat assessments.
- Results
  - So that the reader can better interpret the analysis results, I suggest adding a visual representation of the models’ inter-relatedness in addition to the explanation in the section “Krill growth models and aims of this study” (this can even be a supplementary figure). For example see below. Also ensure that the ordering of the models throughout the figure panels is consistent. E.g. in Fig 1 and Fig 2, the ordering of the models is different.

- Discussion
  - I would like to see an element added to the discussion of the mechanistic model comparison. I was surprised to see in Fig 4 (but also visible in Figs 3 & 5) that the Bahlburg et al 2021 model produces very similar results to Hofmann & Lascara (2000) and Fach et al (2002), despite the Bahlburg et al model being described as more structurally similar to Jager & Ravagnan (2015). Can you elaborate/clarify why this might be?
- Method
  - To clarify the methodology for the pre-screening of models for inclusion of the analysis, it would be good to improve transparency by adding a supplemental figure listing all the models assessed (rows), the criteria required for inclusion in the study (columns), and an indication of which criterion each model passed/failed to result in the suite of models included in the study.
  - Lines 681-686: From the results it is clear that negative growth is possible under certain environmental conditions; therefore why is growth only set to 0 when there is no data for chlorophyll a? Could this not overestimate the growth during these periods? Please explain/justify this methodological choice.
- Code.
  - Overall, the code is very well-organized and easy to interpret, and I commend the authors on their work. I have a few specific questions/suggestions itemized below.
  - README file: I recommend specifying the versions of all the packages used to run the analysis, as well as the version of R.
  - File: 02BahlburgEtAlSummerGrowth.R
    - Line 31: why is initial photoperiod subset to the first layer and not the layer equivalent to startDay (i.e. 306)? Should this line be “initPhoto <- subset(photoClimatologies,startDay)” ? This is likely a minor error because in the loop any following photoperiods are identified by dayOfYear.
      - Check this line for other models that also have initPhoto variable (e.g. 03ConstableAndKawaguchiSummerGrowth.R)
    - Line 101: I suggest to remove line break between “}” and “else{“ so that the line is “}else{“, otherwise the script fails and crashes.
  - File: 08TarlingEtAlSummerGrowth.R
    - Line 43 & 47 : “TarlingEtAl2006IMPRounded” object is not defined in the script, so could not run the rest of the code
  - File: 10ManuscriptPlots.R
    - Code for p3 (lines 87-118): The line for the average trajectory for the Fach et al 2002 model does not match the figure in the manuscript (see below). Please double check the code in File 04FachEtAlSummerGrowth.R

- Line 155: object “modelResultsSub” not defined so modelResultsCV cannot be calculated
- Lines 181-202: A section of the maps appears in the modelDiff plots which appears as “NA” in Fig 5 (see yellow circled region in figure below of the differences from the Atkinson et al 2006 model)
- Line 357: “library(geomtextpath)” needs to be run before p1 can be generated
- Line 429: change to “ggsave('plots/RyabovChla.pdf', plot = p1, width = 7, height = 6)” so that the file path is relative to the working directory (same applies to line 450, 473, 494, 514)
