# Supplementary material for: An intercomparison of models predicting growth of Antarctic krill (Euphausia superba): The importance of recognizing model specificity
Source: PLoS One. 2023 Jul 28;18(7):e0286036. doi: 10.1371/journal.pone.0286036 (PMC10381086; doi:10.1371/journal.pone.0286036)
Supplement: Attachment — Submitted filename: Response to Reviewers.docx [file pone.0286036.s012.docx]

“An intercomparison of models predicting growth of Antarctic krill (*Euphausia superba*): the importance of recognizing model specificity” submitted to PLOS One by Dominik Bahlburg, Sally Thorpe, Bettina Meyer, Uta Berger, and Eugene Murphy

Response to the comments made by the reviewers

We would like to thank the reviewers for their thorough, constructive, and generally positive comments and appreciate their considerable efforts to improve our manuscript.

**Reviewer 1:**

**Main Comments**

Reviewer #1: The manuscript by Bahlberg et al. provides, for the first time, a detailed comparison of Antarctic krill growth models. This is an important study with valuable findings. It is well written, and the analytical approach has been performed at a high technical standard. The details provided in the manuscript are clear and thorough. Model code has been made available to the public for transparency. Overall, I recommend this manuscript for publication with a few minor revisions that I will outline below.
In general, I think the following concepts are not explored in the paper and I recommend that they be brought into the Discussion section somewhere:

**Comment 1:** Not all chlorophyll-a is accessible or preferential to krill. The only real discussion of chlorophyll-a is about total concentrations, but I think it would be pertinent to also talk about the fact that generally under high chlorophyll-a concentrations, diatoms typically dominate whereas smaller phytoplankton often dominate at lower chlorophyll-a concentrations. The latter tends to shift the food web and increase the number of trophic steps before krill – they tend to become more carnivorous. This leads into the next topic that I think the paper would benefit from a brief discussion of.

*DB: Since Comment 1 and Comment 2 are closely related, both are addressed below.*

**Comment 2:** Not all food is equal in terms of growth. We know from previous lipid studies that krill need diatoms and some protozoans for growth. If krill aren’t eating diatoms, but are still getting a lot of food through carnivory, they might not be growing as fast or as much.

*DB: We agree that the accessibility of different primary producers for krill as a food source as well as their dietary value as a function of lipid content, lipid composition, body size, and functional group are important issues that had not been addressed in our discussion.*

*These issues also highlight the dichotomy between the empirical side of krill ecology and its modeling. While these aspects of krill ecology are obviously highly relevant, they are also something that will be extremely difficult to incorporate into large-scale model simulations in the near future. Not only do we lack adequate data to robustly parameterize model functions that predict food quality as a function of lipid content, body size, and functional group of the food source, but data sets that estimate these environmental variables at sufficiently large temporal and spatial scales are not currently available (although Fach et al. (2008) introduced a first krill growth model that incorporated the nutritional composition of different food sources).*

*We now included these important aspects in a new paragraph in the subsection “Towards more general krill growth models” in the Discussion.*

**Comment 3** I was anticipating the manuscript mentioning which of the models seemed to be the most accurate. This doesn’t happen in the paper though and I think it would be helpful to have some discussion about whether there is a “best” model. Are the mechanistic models better than the empirical models or the other way around? It feels a bit like you have to read between the lines to decipher this. If it is not yet possible to say which is better, then I think this should be stated so that the reader isn’t left wondering.

*DB: We wish we could have made a recommendation or ranking of the best model, but given the results of this study, we have refrained from doing so. 1. We did not want to give the false impression that some models should be used and others should not. Each model has its validity and has demonstrated its ability to realistically reproduce empirical krill growth and/or other life history data at the time of its introduction. 2. Given the limitations and specificities of each model, each could be the best candidate, depending on the spatial, temporal and ecological context, as well as the specifications of the environmental input data; and for some combinations none may be appropriate. This is now more explicitly addressed in the conclusion:*

*“When the models are intended to be used with input data that systematically deviate from their calibration data (e.g. satellite data vs. in-situ data), they need to be re-calibrated for the respective data type. There is no best or worst model, but rather the regional and temporal context of a simulation study, as well as the technical specifics of the input data, largely determine the most appropriate model candidate. Nevertheless, it also becomes clear that uncertainty in the relationships underlying fundamental model building blocks, such as between temperature, food availability and growth rates leads to large uncertainty in model simulations. As with the use of multiple models to predict future climate scenarios, simulating the response of krill to environmental change by using different models in parallel could be used as an opportunity to learn more about our understanding of how the system works, and ultimately lead to more reliable predictions, not in spite of, but because of the explicit consideration of model uncertainties. We hope that our model library and open access code can contribute to this journey.”*

**Comment 4** Title: After reading the manuscript, I came to the conclusion that the title might be a little misleading. In particular, the part “towards a generalised understanding” suggested to me that there would be a generalised understanding of Antarctic krill growth, but I’m not convinced that is necessarily achieved here, nor am I sure that was the goal. It seems to me that the goal of the manuscript was to compare the various modeling approaches and make recommendations on how best to proceed in the future. I think the paper does this well. I would recommend slightly changing the title to something along the lines of “Intercomparison of growth models for Antarctic krill (Euphausia superba): towards a generalised approach” or “…towards a common approach” or something like that, which more clearly articulates the outcome of the paper.

*DB: We agree that the original title did not fully reflect the work presented in the manuscript and changed it to “An intercomparison of models predicting growth of Antarctic krill (Euphausia superba): the importance of recognizing model specificity". In doing so, we also hope to make an important finding of this study more explicit (model specificity).*

**Comments referring to specific lines in the manuscript:**

**Line 19:** “…the most dominant…” – The word “dominant” has different connotations and “abundant” would be a better, more precise term here.

*DB: changed the “dominant” to “abundant” and made slight adjustments in the previous part of the sentence to reduce redundancy*

**Line 28:** I would rephrase to “Growth rates of individual krill…”

*DB: changed to “growth rates of individual krill”*

**Line 29:** Add a comma before “which”

*DB: added comma*

**Line 124:** Delete “well” from “…were not well compatible…”

*DB: The sentence is now rephrased to “The dynamic optimization models of Alonzo and Mangel (2001) and Richerson et al. (2015) are backward-in-time models that optimize krill life-history decisions to maximize fitness, and thus did not fit with our research goal of evaluating models in the context of predicting krill growth potential and habitat quality under current and future environmental change.”*

**Lines 138-139:** Correct sentence to “…was reduced in such a way as to make…”

*DB: we corrected the sentence accordingly*

**Line 186:** Add a comma before “which”

*DB: added comma*

**Line 187:** Add a comma before “which”

*DB: added comma*

**Line 189:** Because of the way it is written, it would be helpful to include the author name in the citation of “[68]” – i.e., “…from experimental work by Piccolin et al. [68]”. I noticed that this was sometimes done and other times not. I will include notes on the ones I caught, but I don’t think I noted it down in every instance so please go through the entire manuscript to make sure where you refer to a paper within a sentence as in the above example, you include the author’s name.

*DB: We now added the author names in in-line references throughout the manuscript to improve readability.*

**Line 192:** This is another example of where I think you could include the author’s name – “…described by Tarling [69]” – also note that it should be “by” rather than “in”.

*DB: see comment above*

**Line 204:** Add a comma before “which”

*DB: added comma*

**Line 238:** Change to “growth to these key environmental variables”

*DB: The text is now changed accordingly*

**Lines 282-283:** “operation” is used twice in the sentence. You can delete the second occurrence.

*DB: This paragraph is now restructured based on comments made by Reviewer 2 and “operation” no longer appears in the sentence.*

**Lines 292-293:** “change” is repeated twice in this sentence. You can delete the second occurrence. Also, this sentence is still quite hard to read/follow. Perhaps consider rephrasing for further clarity.

*DB: Based on your comments and those made by Reviewer 2, this text passage is now rephrased to “The five mechanistic models, on the other hand, are insensitive to chlorophyll a concentrations <1 mg m^-3^ (visible as broad areas of low growth rates of <0.1 mm d^-1^) and require higher food concentrations for more rapid growth (see S3 Fig) which shows chlorophyll a concentrations required by each model to predict growth rates of 0.1 and 0.15 mm d^-1^). As a result, growth rates with increasing chlorophyll a are generally lower, as are maximum growth rates.”*

**Line 356:** Add a comma after “expected”

*DB: added comma*

**Line 371**: Add a comma before “which”

*DB: added comma*

**Lines 373-374:** This statement is made several times in the paper, but it’s not explained whether this is an incorrect assumption or not. I think it would be helpful to the reader if this was expanded on.

*DB:* *In this context (and as acknowledged in your comment on lines 554-555), this statement is not necessarily an assumption, but a direct consequence of the model structure, because with positive instantaneous growth rates (as specified in the text), more frequent moulting directly corresponds to more frequent increments in body length, resulting in higher overall growth rates. But we now expanded on this in Lines 554-555 (see comment below)*

**Lines 511-512:** Change to “…specific to certain regions and seasons, and constrained to…”

*DB: we adjusted the text accordingly*

**Line 517:** Remove the space between “-“ and “comparison in “cross- comparison”

*DB: we removed the space*

**Line 525:** Add the author name, i.e., “For example, Murphy et al. [29] considered…”

*DB: see response to previous comment. In-line references are now referred to by the author name throughout the manuscript.*

**Line 552:** Change to “Another example of…”

*DB: we changed the text accordingly*

**Lines 554-555:** I think this statement is an oversimplification. More frequent moulting could also occur if the krill need to shrink rapidly, which would not lead to higher growth rates. However, more frequent moulting and a positive growth increment will result in higher growth rates.

*DB: This is true, and we now included an additional sentence in the Discussion to clarify this. However, the instantaneous growth function of Atkinson et al. (2006), which is used by the models of Tarling et al. (2006) and Wiedenmann et al. (2008), predicts positive growth for large areas at most time points in our circumpolar simulations (which did not include less productive periods such as late fall or winter). The additional sentence is*

*“Another example of uncertain mechanisms are the two intermoult period-based models [31, 52] that predict growth as an increment in body length at the event of moulting. For the model dynamics, more frequent moulting implies higher growth rates whereas fewer moulting events correspond to less growth, when growth rates are positive. When growth rates are negative (e.g. in winter, which we did not address in this study), more frequent moulting would result in rapid shrinkage.”*

**Line 561:** I am not sure what you mean by “reversely interpreted”, please rephrase this for clarity.

*DB: The entire paragraph is now rephrased to:*

*“The two intermoult-period-models differ considerably in the range of values each predicts and the relationship between intermoult period and temperature. For example, predicted intermoult periods are predicted to increase at temperatures >0.5 °C by the model of Tarling et al. (2006) whereas they decrease in the model of Kawaguchi et al. (2006) (using the "adult female" parameterization of the model of Tarling et al. (2006), S2 Fig; for "juveniles, the temperature-intermoult period relationship is slightly differently shaped with a maximum at 3 °C and lower values at lower and higher temperatures, respectively).”*

**Lines 589-593:** See my second commend under the broad comments before the line-by-line comments. I think some discussion on the role of different food types in promoting and supporting growth is needed.

*DB: A new paragraph is now added to the discussion to touch on other important aspects of krill feeding ecology that is not captured by the models and simulation.*

**Line 624:** Please write ODD out in full here.

*DB: we changed the text accordingly*

**Lines 640-654:** There is no mention on the effects of clouds for ocean colour and this should probably also be included, even just briefly.

*DB: We completely agree with your statement. However, the dataset we used is an "L4"-processed dataset, which means that data gaps caused by cloud cover are extrapolated by different algorithms, meaning that cloud cover had no effect on the availability of data points for our simulations. We have made this detail more explicit in the text now:*

*“The data were reprocessed and interpolated to fill gaps that can be introduced e.g. by cloud cover (the full processing and a quality assessment of the dataset is available under https://doi.org/10.48670/moi-00281)”*

**Line 685:** How did you choose 1 day?

*DB: We chose 1 day as the integration time step because it allowed the numerical integration schemes to be sufficiently accurate. The numerical approximation/integration of the models becomes more accurate the smaller the time step, at the expense of computational time. Using too large a time step results in significant numerical artifacts during the integration of the structurally complex models, which often leads to results that approach infinity. When implementing the models, we found that timesteps smaller than 1 day would not have changed the results, but would have required significantly more computation time. In addition, a time step of 1 day conveniently matched the temporal resolution of the environmental datasets. This additional information is now also included in the Methods as “The time step of 1 day ensured sufficient precision in the numerical integration of the models and matched the temporal resolution of the environmental datasets.”*

**Line 730:** Include author name, as in “…model of Constable et al. [64]…”

*DB: we now included the author name here, and throughout the manuscript for in-line references*

**Lines 740-742:** What are the “respective Figures”? Please rephrase for clarity.

*DB: We now specified the sentence to “We extracted the environmental data from the corresponding figures in the original studies in which each model was presented, using the data extraction tool from ImageJ”*

**Figure Captions:**
**Figure 3.** caption on page 7: I would add “(black circle) after “15th April” to be consistent with the mention of “(white circle)” following “1st November”.

*DB: we added “black circle” after 15^th^ April*

**Figure 4**. caption on page 9: There is a floating, unused “(“ on the fifth-to-last line.

*DB: The floating “(“ was due to the missing reference “and Tarling et al. (2006)”, which has now been added.*

**Figure 5.** caption on page 10: Remove the “.” after “1st” and before “November”

*DB: removed the “.”*

**Reviewer 2:**

**In-line comments in the pdf file:**

**Abstract:** I suggest here to use a more general term such as "compare" -- cross validation can refer to a very specific form of model testing & selection (e.g. in machine learning applications).

*DB: Changed to sentence to “The available models have been developed using a range of empirical and mechanistic approaches, providing alternative perspectives and comparative analyses of the key processes influencing krill growth.”*

***Author Summary Comment 1:*** and extrapolated?

*DB: added “and extrapolated”*

**Author Summary Comment 2:** I think to connect to some of your key points from the next sections, perhaps so re-word this because it seems that it's not necessarily that the models are applied across different scales, but more so that the models have been developed using data from specific points and in space and time and it is unclear to what extent they can be used for extrapolation?

*DB: changed text to “There are a number of krill models that have been developed using data from different spatial and temporal scales, and it is unclear to what extent they can be extrapolated and how model-dependent the results and their interpretation are.”*

**Line 8:** "these relationships" -- I think a relationship quantifies how the independent variable (environmental stressor) impacts the response, so response is already included

*DB: changed to “these relationships.”*

**Line 8:** Biophysical models? Because this is referring to models at different scales (not just ecosystem)

*DB: changed to “Biophysical models”*

**Line 34:** .. and how it can be inferred from length-at-age relationships

*DB: changed to “and how it can be inferred from length-at-age relationships”*

**Lines 37-38:** Reference?

*DB: Added Thorpe et al. (2007) and Piñones et al. (2013) as references as these studies demonstrate the potential connectivity of krill populations from local to circumpolar scales.*

**Lines 69-74:** **Comment 1:** I think another sentence of elaboration is needed here to fully understand how the approaches are complementary (i.e. the different insights they provide).

*DB: See reponse beneath Comment 3.*

**Comment 2:** I suggest to add another advantage -- that within the bounds of the data to which they were fit, the empirical models can provide predictions without requiring a complex, precise knowledge of the underlying ecophysical mechanisms.

*DB: See reponse beneath Comment 3.*

**Comment 3:**

What is meant by "potentially increased generality"? Under what instances would this occur? I think saying generality alone here might be inaccurate -- you could (theoretically) have a mechanistic model which is finely tuned using data from an individual krill which does poorly at representing the entire population. I would say instead that they can provide precise predictions while having an increased ability to extrapolate predictions to new environmental conditions because the underlying mechanisms are represented

*DB: We rephrased the paragraph to address the three comments:*

*“Rather than explicitly including krill growth mechanisms, the empirical models were developed by correlating observed growth rates to environmental conditions in the southwest Atlantic and Indian sectors of the Southern Ocean. The advantages of the empirical model approach are its computational efficiency, its close connection to observations and its ability to make predictions without requiring precise knowledge of the underlying ecophysiological mechanisms, within the limits of the data to which they were fitted. The main advantage of the mechanistic approach is the explicit inclusion of ecophysiological mechanisms. This arguably improves the ability to predict krill growth under environmental conditions other than those to which the models have been fitted, as the underlying mechanisms of growth are represented in the models. As identifying, characterising and parameterising the mechanisms of growth can be very challenging in practice, having the two approaches is valuable as they provide different perspectives and the opportunity for comparison of the outcomes.”*

**Line 100:** Is the range specifically what was compared? I would remove this and just say "compared"

*DB: Removed “the range of”*

**Line 104:** Again "generalizable" seems vague. I would even reword "Our results will contribute towards the development of krill growth models that are better suited for extrapolation in environmental space, particularly under future change"

*DB: changed sentence to “Our results will contribute to the development of krill growth models that are better suited for extrapolation in environmental space, particularly under future change*”

**Line 112:** only one criteron (at least one environmental input variable) -- change from the plural "these criteria"?

*DB: changed to “this criterion”*

**Fig. 1:** In other figures, an asterisk is used to indicate empirical model types -- for consistency I suggest to add here as well

*DB: We now used asterisks to highlight the empirical models in all Figures as well as in the Tables.*

**Line 120:** To streamline this, why not combine all these eligibility criteria and present the results in one step? A table could be provided in the supplement with a list of all models considered (rows) crossed with the different criteria (columns), and indications of whether each model failed or passed each criteria. The models that pass all criteria can be presented in the main text (i.e. present Fig 1 above the black line).

*DB: This was a useful suggestion, thank you, and we have now included a table in the Supplementary Material illustrating the model selection process. However, we kept the previous brief description of the model selection in the main text. Including the table summarising the model selection process in the main text (instead of the appendix) would probably take up at least as much space as the current text (at least 17 lines for each of the models + padding plus a short description vs. the current 19 lines of text).*

**Lines 124-125:** ... compatible with our research aim of evaluating models in the context of predicting habitat under current and future environmental change?

*DB: changed sentence to “The dynamic optimization models of Alonzo and Mangel (2001) [63] and Richerson et al. (2015) [64] are backward-in-time models that optimize krill life-history decisions to maximize fitness, and thus did not fit well with our research goal of evaluating models in the context of predicting krill growth potential and habitat quality under current and future environmental change.”*

**Line 136:** Later on it is stated that Wiedenmann et al (2008) uses the growth increment of Tarling (2006) -- which is it/or is it both?

*DB: Thank you for spotting this inaccuracy. The model of Wiedenmann et al. (2008) combines the IMP-function of Kawaguchi et al. (2006) with the instantaneous growth function presented in* ***Atkinson et al. (2006).*** *Therefore, we corrected the “Tarling et al. (2006)” to “Atkinson et al. (2006)” in line 265.*

**Line 139:** This paragraph is a bit confusing -- above it reads as if Atkinson et al (2006) also solves for IMP

*DB: We hope that we clarified the confusion by adjusting the previous paragraph to make the model structures clearer. It now reads as:*

*“The models of Atkinson et al. (2006) and Tarling et al. (2006) are empirical models that were generated by correlating observations of growth rates and IMPs with water temperature. Although not a standalone growth model, the model of Tarling et al. (2006) describes IMP functions that were used to develop the instantaneous growth model in the study of Atkinson et al. (2006), which is based on data from the same cruise and region. The model of Wiedenmann et al. (2008) is a composite empirical model that combines the instantaneous growth functions from Atkinson et al. (2006) with the IMP model of Kawaguchi et al. (2006). In the development of the model of Wiedenmann et al. (2008), food availability was reduced in such way as to make the model reproduce growth curves presented in Siegel (1987). To test an alternative parameterization to the model of Wiedenmann et al. (2008), we combined the IMP functions of Tarling et al. (2006) with the growth functions of Atkinson et al. (2006). The results from these simulation runs are and labelled "Tarling et al. (2006)" in the Figures.*

**Line 158:** I like this figure -- it does a good job of summarizing the models used

*DB: Thank you, that is nice to hear.*

**Line 225-233:** This may belong in the methods or discussion section because it doesn't present a result per se, but more so discusses the rationale and limitations of this methodological choice

*DB: We agree that the Discussion might be a better place for this and shifted the text to the “Towards more general krill growth models” subsection of the Discussion*

**Line 235:** remove

*DB: removed “ranges of”*

**Line 268:** This is difficult to interpret from the plot, because it appears the colour bar ends at 0 -- can more changes in colour scale be added/the colour scale bar extended so that this is perceptable?

*DB: We agree and also apologize for the inaccuracy of hiding negative values in the colour scale. The colour scale now spans over the full range of daily growth rates with red colours for negative, and blue colours for positive growth rates. We did the same for the growth plane Figure contained in the Supplementary Material (S2 Fig)*

**Lines 282-283:** The repetition of the word "operation" here makes the sentence a bit confusing to the reader

*DB: After reading this passage in the context of Figure 3, we decided to change this sentence entirely. The seasonality of growth in the mechanistic models is an important component driving the differences shown in Figures 4 and 5, but not in Figure 3, which compares predicted growth rates for the same day of the year. The paragraph now begins as:*

*“The main reason for these differences is that the models were built and calibrated against very different food availability datasets from different locations, seasons, and numbers of observations. As a result,[…]”*

**Line 292:** increase in growth rates with increasing chlorophylla a being ...

*DB: changed sentence to “As a result, growth rates with increasing chlorophyll a are generally lower, as are maximum growth rates.”*

**Line 295:** can be more precise -- "over the ranges of chlorophyll a and temperature assessed".

*DB: changed sentence to “The models of Bahlburg et al. (2021) and Jager and Ravagnan (2015) predict the lowest growth rates over the ranges of chlorophyll a and temperature assessed.”*

**Fig 4:** Because the range of the environmental data predicted involves extrapolation for some empirical models, this limitation can be highlighted by changing the line colour (or a similar visual cue) if at least one of the environmental variables is outside the range of the empirical data

*DB: Except for the first 4 weeks of the simulation when water temperatures were between -1.5 and -1* °*C, the range of temperatures and chlorophyll a concentrations in the environmental time series at the location close to the South Orkney Islands falls within the individual ranges of temperature and chlorophyll a concentrations that the instantaneous growth functions and daily growth rate functions in Atkinson et al. (2006) were fitted for (see Table 1 and Fig. 5 in Atkinson et al. (2006); Figure 8 in Tarling et al. (2008)). The IMP model of Kawaguchi et al. (2006), which is used in the model of Wiedenmann et al. (2008), was fitted and tested for temperatures as low as -1*.8 °*C.*

*Therefore, changing the line colours of the empirical models for the first 4 weeks of the growth trajectories is not very informative. However, we have now added a sentence to the text describing the figure pointing out this feature.*

**Fig. 4:** Same comment as above for Fig 4 -- is it possible to add a contour line on the maps of the empirical model simulations so that it is clear where the models are being extrapolated?

*DB: Thank you for this suggestion. We tried to address this comment in several ways, e.g. by*

- *creating new maps for each model, counting the days in each grid cell where at least one environmental parameter was outside the range used in the model development*
- *drawing contour lines for regions where environmental conditions were outside the data range for a minimum number of days (e.g. 25% of the simulation period).*

*However, the results were not very convincing. As Fig 5 is already very information-rich, and as the extrapolation of models to data outside the range used for their development is discussed frequently throughout the manuscript, we decided not to follow this suggestion. We hope you understand our decision.*

**Line 336:** *add “mechanistic”*

*DB: added “mechanistic”*

**Lines 433-435:** If this information is key to your discussion, then can this be added into Fig 1 for example? Perhaps a different colour when an environmental input variable is derived from remote sensing or other?

*DB: The information whether chlorophyll a data were satellite derived or not is now added as an additional colour to the Table and an explanation was added to the Caption.*

**Line 485:** breifly list "such as ... (reference)"

*DB: added “such as the Scotia Sea […]”*

**Lines 498-500:** Add more precision to this statement -- which areas/models in particular?

*DB: added more precision: “While some models generate negative rates, others predict more reasonable rates for areas where high growth rates are expected, such as in the Scotia Sea when comparing the model of Jager and Ravagnan (2015) with the model of Ryabov et al. (2017) or Bahlburg et al. (2021).”*

**Line 516:** I agree with the thought here but think combining "regional specificity" and also the word "generality" creates confusion because they can seem to be contradictory. Also is the text referring to one general model, or a general suite of models, or a suite of specific models which together acheive generality?

If I understand the statement here, this study has assessed a suite of models (whose input data vary in space and time) in terms of their suitability for extrapolation to model krill growth at larger spatial scales (across their circumpolar range) and temporal scales (over the entire seasonal cycle). A main finding is that models perform well within the bounds of the data to which they were parameterized. Thus, in order to accurately predict growth across the circumpolar range of krill, accounting for regional and seasonal variability in the model parameters is important. Models calibrated using data from one region/season are not very generalizable on their own. To increase generality, this can be done within the framework of one model by re-calibrating it using data with a larger coverage and modelling the mean population response while reducing specificity; or by using a suite of specific models that complement each other to acheive full spatial and seasonal coverage.

*DB: These are good points and after re-reading the paragraph we agree that it needs to state more clearly what the potential outcomes of an extensive model cross-comparison could be. Therefore, we now listed them as:*

*“Such effort could potentially lead to three outcomes:*

1. *region-specific parameterizations of single models (individual model, regionally adaptive parameters)*
2. *region-specific models, whose predictions are combined for large-scale assessments (complementary approach)*
3. *models (individual models or ensemble) that predict the mean response of krill in large-scale assessments, incorporating the regional variability of observations as variance around the mean*

*Future studies to evaluate these approaches will be an important next step in this research.”*

**Line 633:** Can these datasets be cited in the references (in addition to providing the dois in text)?

*DB: The datasets are now referred to by their common names and references were added.*

**Line 643:** remove period?

*DB: removed period*

**Lines 652-653:** Were there any cells in which the mean value was determined by a relatively low proportion of years? E.g. Across the whole climatology, say there was 1 non-NA value in a grid cell. Effectively the mean would be equal to the value of the one cell. If so, was this bias adressed?

*DB: Grid cells with climatologies based on a small number of samples sometimes occur in regions that were ice-covered in most, but not all, years. The maps below show the average number of observations available to compute the climatologies (before applying the 15d moving average) in each of the simulated months (in the case of April, only until April 15th, in accordance with the simulation setup). It can be seen that regions with low sample numbers (but >0 observations) are relatively widespread in November but much less so in the other months. In the second Figure of this comment, we visualized the chlorophyll a concentrations predicted for low-sample regions (<4 samples for climatologies). For the vast majority of these regions, the average chlorophyll a concentrations are <0.5 mg m^-3^, which means that the predicted growth rates in these regions are low, consistent with the zero-growth assumption for ice-covered areas.*

*We acknowledge that, particularly in November, these low sample regions may slightly bias the chlorophyll a climatology and consequently the model predictions, but the effect is limited in time, small in magnitude and applies equally to all models. Therefore, these biases probably negligible for the purpose of this study, which primarily focused on comparing growth models when simulated under the same environmental conditions, not to maximize the realism of the predictions.* *In addition, all of the biases applied in the same way to all of the models, which, most importantly, makes the results directly comparable. In addition, for comparing growth trajectories (north of South Orkney Islands, Figure 4), we chose a location that is typically ice-free.*

*To be more transparent, we now included the following statement in the Methods section: “Regions where climatologies were based on a low number of samples (e.g. less than 4) occurred predominantly in November. Chlorophyll a concentrations in these regions were usually <0.3 mg m^-3^ meaning that the predicted growth was low, consistent with the overall growth patterns at this time of year. The slight biases introduced by this applied equally to all growth models, which still allowed for a meaningful intercomparison, which was the main purpose of this study.”*

**Line 665:** I found the placement of the reference to Appendix S1 File a bit awkward -- it seems like a step that happens before the simulation is run, so I think it needs to be further up/even at the beginning of this section

*DB: We now placed the “Model implementation” subsection to the top of the Methods section. Therefore, the reference to S1 File now appears at the beginning of the Methods.*

**Line 701:** Check for the directions of the quotation marks

*DB: The manuscript is rendered from a LaTex file with PLOS-specific typesetting formatting. I think the direction of the quotation marks is something that is either PLOS specific or needs to be addressed by the final typesetting done on the PLOS side. All quotation marks that occur in the manuscript have this issue.*

**Line 713:** Not sure why Appendix File S1 is referenced here? Fig S4 seems to support this statement

*DB: Thank you for pointing this out, the reference to Appendix File S1 is now removed.*

**Line 728:** I suggest for clairity, move this section before the description of the simulations run

*DB: We agree with this suggestion and moved this sub-section to the beginning of the Methods section*

**Line 777:** Update the README.md file in the github repo with this link for the input files so it is stand-alone

*DB: The README.md is now updated and the link added.*

**Comments made in the Supplementary Material**

**Comment 1:** I suggest adding a reference list to the supplement that is independent of the main manuscript -- otherwise ensure to cite the numbers of the references that match those in the main manuscript reference list

*DB: An independent bibliography is now added to the Supplementary Information*

**Comment 2:** Reference?

*DB: Reference for ImageJ is now added*

**Comment 3:** Hofmann and Lascara (2000) -- just saying the paper is confusing because it could refer to the main manuscript of this publication

*DB: replaced “paper” with “Hofmann and Lascara (2000)”*

**Comment 4:** remove

*DB: removed “actually”*

**Comment 5:** Language here may be too colloquial -- suggest re-wording (e.g. Upon consultation of the Fortran files, the reason for this discrepency between our implementation and the results reported in Hofmann and Lascara (2000) could not be identified).

*DB: We have reworded the entire paragraph, as we felt that it contained information that was confusing rather than informative. In doing so, we also removed the colloquial language. It now reads as follows:*

*“Including ice algae as a second food source results in stronger differences between our implementation and the originally reported trajectories. Growth is strongly overestimated in our version. In our project, we restrict the simulations to summer growth and excluded ice algae as a food source - also because no circumpolar predictions of sea ice biota (accessible for krill) exist. Therefore, potential inaccuracies in the circumpolar model simulations are comparable with those shown in the left panel of Figure 1.”*

**Comment 6:** This seems to contradict a sentence later in this paragraph which states that temperature-dependent metabolic cost is the only change? Here it reads as if there are two changes.

*DB: From our point of view, adding heterotrophic is not really a structural difference between the models since in the model of Hofmann et al. (2000), chlorophyll a is converted into carbon using a conversion constant before the budget and energetic costs are calculated. Adding heterotrophic food as a food source is therefore no structural change in the model but just another carbon value that is added to the phototrophic and ice-algae carbon. Since we excluded heterotrophic food from our simulations due to the difficulties of appropriately parameterizing it, the temperature dependence of metabolism is the only structural difference between the two models in our simulations. We agree that this may not have been clear before, and we have now made this more explicit with the following statement:*

*“As the modifications made to the original model are rather minor (with the exclusion of heterotrophic food in our simulations, both models are structurally identical except for the temperature dependent metabolism in Fach et al. (2002)), we assume that the quality assessment yields similar results as the one on Hofmann and Lascara (2000).”*

**Comment 7:** Could you elaborate more on why? Is this because the environmental data are extracted along the advected trajectories of the particles but are only presented in the figures in the paper as extracted data along the trajectories of several drifters? Because otherwise the datasets appear available (e.g. for chlorophyll a : <https://oceancolor.gsfc.nasa.gov/data/czcs/>)

*DB: We double-checked the manuscript and again concluded that it is not possible to fully reconstruct the environmental conditions used to generate the growth trajectories, e.g. shown in Figure 8b (phytoplankton as the only food source, analogous to how we applied the model). According to the authors, the chlorophyll a concentrations used for the simulation run are shown in Figure 3D (drifter 1), but it is unclear what the corresponding temperature data were, and it is also surprising that rapid changes in chlorophyll a concentrations in Figure 3D, such as around day 80 (day of the year), where they suddenly increase from almost 0 to 1.5 mg m-3, are not reflected in the corresponding growth trajectories. Since we have validated the implementation of the model of Hofmann and Lascara (2000), which is the baseline model for Fach et al. (2002) except for the addition of other food sources that we did not consider and a Q_10_ function that relates metabolism to temperature, we believe that the model is reproduced well enough to include it in our general model comparison. In our simulations, the model produces results that are very similar to the model of Hofmann and Lascara (2000), which is also in good agreement with the simulation results shown in the original publication of Fach et al. (2002), where the temperature scaling function had only minor effects on krill growth (krill reached final body lengths that were 0.5 mm larger in the temperature-dependent model compared to a control run).*

*However, going back to the model and its code revealed a minor inaccuracy in our old implementation. Growth rates in the Fach et al. (2002) model are capped at 0.25 mm/d according to the original study. However, in the previous version we applied the temperature scaling of growth* ***after*** *the growth rate was capped, meaning that under certain environmental conditions* ***growth could exceed the maximum growth rate if the Q10 equation returned values >1****. This has now been corrected, and the temperature scaling is applied to respiration and ingestion rates before net production is calculated. As a result, the shape of the growth rate curves shown in Figure 3 is now slightly different for the Fach et al. (2002) model, especially at high chlorophyll a concentrations where growth rates >0.3 mm/d were previously possible due to the issues outlined above. Since the environmental conditions in our simulations rarely allow for high growth rates in the model, this modification has no effects on the general Discussion and neglectable effects on the results shown in Figures 4 and 5.*

**Comment 8:** Double-negative here was a bit confusing -- suggest stating as "it is likely that we have implemented it correctly"

*DB: changed the sentence to “it is likely that we have implemented it correctly”*

**Comment 9:** Ensure that the supplementary figures presented in the appendix are still referenced -- at the start of this paragraph it was confusing as to which figure it was referring to (Fig 9 or 10 in this file).

*DB: The Figures included in the Supplementary Material are now referenced in this section.*

**Comment 10:** Is this referring to the black line on the bottom left panel in Fig 9 of this Appendix? If so, it appears the black line does go back to zero when the red dotted line does -- please explain

*DB: We fully agree with your comment and appreciate your rigor. This was an incorrect statement and it has now been removed.*

**Comment 11:** There appears to be no top or bottom panel because the two panels are side by side?

*DB: This is correct, we had the wrong figure width settings for the two sub-Figures in the LaTex document. This has now been corrected so that the Figure consists of a top and bottom panel similar to Figure 9.*

**Main Comments of Reviewer 2 from the included docx-File**

**Introduction**

- When using the word “generality” in terms of the analysis aims, I think this needs an extra sentence or two to provide a more explicit definition. The way “generality” appears to be used here refers to how suitable a model is for extrapolation in geographic space, in time, and in environmental space (i.e. outside the environmental conditions it was calibrated for). By extrapolating models across these different dimensions, it is possible to validate the model predictions with existing knowledge on krill distribution and habitat quality. This helps define the limits of how a model may be suitably applied for of current and future habitat assessments.

*DB: This is a very good suggestion and we now included parts of it in a new paragraph in the subsection “Krill growth models and aims of this study”:*

*“In our study, we assess the structures and limitations of these models, seek to understand the differences, and consider the generality between them. Generality in this context is assessed in two ways: 1. It refers to the way in which a given model is suitable for extrapolation in geographic space, in time, and in environmental space (i.e., outside the environmental conditions for which it was calibrated). 2. Generality also refers to the differences in predictions between models when simulated under the same environmental conditions. If the models evaluated in this study predict comparable results, it means that the mechanisms of krill growth (or their statistical approximations in the case of empirical models) are general and hold across a wide range of habitats and seasons, since the models originate from different regions and time periods.”*

**Results**

- So that the reader can better interpret the analysis results, I suggest adding a visual representation of the models’ inter-relatedness in addition to the explanation in the section “Krill growth models and aims of this study” (this can even be a supplementary figure). For example see below. Also ensure that the ordering of the models throughout the figure panels is consistent. E.g. in Fig 1 and Fig 2, the ordering of the models is different.

*DB: We have now added a new Figure called “Lineages of krill growth models” to the Appendix and refer to it in the main text. The Figure visualizes the chronology, different lineages and interrelatedness of krill growth models developed in the past 50 years (see below)*

**Discussion**

- I would like to see an element added to the discussion of the mechanistic model comparison. I was surprised to see in Fig 4 (but also visible in Figs 3 & 5) that the Bahlburg et al 2021 model produces very similar results to Hofmann & Lascara (2000) and Fach et al (2002), despite the Bahlburg et al model being described as more structurally similar to Jager & Ravagnan (2015). Can you elaborate/clarify why this might be?

*DB: This is a good point and was surprising to us too. The main reason is the difference in the functions used for temperature-dependent scaling of metabolism (Figure 3) as well as the impact of photoperiod on metabolism (Figure 4 and 5). Due to these differences, the energetic demand of the individual krill is much higher In the Jager and Ravagnan (2015) model compared to Bahlburg et al. (2021). Therefore, the growth rates in Bahlburg et al. (2021) are generally higher. The fact that the model of Bahlburg et al. (2021) produces similar results to Hofmann and Lascara (2000) and Fach et al. (2002) is coincidental, since the data and functions used to parameterize the temperature and photoperiod dependence of metabolism (which is indirectly included as a seasonal activity factor in the models of Hofmann and Lascara (2000) and Fach et al. (2002)) are different between the models.*

*This is now added in the Discussion as:
“Another interesting result is that the model of Bahlburg et al. (2021) predicts similar growth as the models of Hofmann and Lascara (2000) and Fach et al. (2002*, *despite being closely related to the model of Jager and Ravagnan (2015). The addition of photoperiod and temperature (using empirical data) as drivers of krill metabolism strongly reduces the energetic requirements of krill individuals in the model of Bahlburg et al. (2021) compared to the model of Jager and Ravagnan (2015), leading to a lower impact of spring and fall starvation periods, particularly in high-latitude habitats, and allows for more efficient ingestion of food during the summer.”*

**Methods**

- To clarify the methodology for the pre-screening of models for inclusion of the analysis, it would be good to improve transparency by adding a supplemental figure listing all the models assessed (rows), the criteria required for inclusion in the study (columns), and an indication of which criterion each model passed/failed to result in the suite of models included in the study.

*DB: We now included a table in the Supplementary Material illustrating the model selection process*

**Lines 681-686:** From the results it is clear that negative growth is possible under certain environmental conditions; therefore why is growth only set to 0 when there is no data for chlorophyll a? Could this not overestimate the growth during these periods? Please explain/justify this methodological choice.

*DB: The reason for setting growth to zero in the absence of chlorophyll a data is a simplified assumption made in the absence of better information. In general, sea ice is considered a food-poor habitat (Meyer et al. 2017) and low to negative growth rates would be expected. On the other hand, especially during the Austral spring and near the marginal ice zone, phytoplankton blooms induced by melting sea ice could lead to comparatively productive regions near, and possibly in under-ice habitats. We agree that this would be an important issue if the ultimate goal of this project had been to maximize the realism of habitat quality predictions, but as argued in a previous response, this was not necessarily the scope of this paper and the same biases applied to all models.*

*We attempted to minimize the impact of this bias on our analysis by restricting the simulations from spring to fall to avoid extensive periods of sea ice cover in important key krill habitats, such as the Antarctic Peninsula or the South Orkney Islands. Similarly, we focused on comparing growth trajectories from regions that were ice-free in our simulations (Figure 4), and emphasized comparisons of predicted krill habitat quality in regions where sea ice concentrations were also generally low from spring to fall (Scotia Sea and Antarctic Peninsula in Figure 5).*

**Code**

- Overall, the code is very well-organized and easy to interpret, and I commend the authors on their work. I have a few specific questions/suggestions itemized below.
- README file: I recommend specifying the versions of all the packages used to run the analysis, as well as the version of R.

*DB: We now used the “renv”-package to create an “renv.lock”-file in the repository, which contains a screenshot of the entire R session and package versions under which the code was developed and successfully compiled. The “renv.lock”-file can be manually opened, e.g. with a basic text editor and the package versions manually looked up. More conveniently, the R environment can be restored/reproduced by simply running “renv::restore()” when the project is opened (see* [*https://rstudio.github.io/renv/articles/renv.html*](https://rstudio.github.io/renv/articles/renv.html)*).*

**File: 02BahlburgEtAlSummerGrowth.R**

- Line 31: why is initial photoperiod subset to the first layer and not the layer equivalent to startDay (i.e. 306)? Should this line be “initPhoto <- subset(photoClimatologies,startDay)” ? This is likely a minor error because in the loop any following photoperiods are identified by dayOfYear.
- Check this line for other models that also have initPhoto variable (e.g. 03ConstableAndKawaguchiSummerGrowth.R)

*DB: Thank you for spotting this minor error (as you mentioned, it does not really distort the simulations). It has now been corrected in both files* (*02BahlburgEtAlSummerGrowth.R and 03ConstableAndKawaguchiSummerGrowth.R*)

- Line 101: I suggest to remove line break between “}” and “else{“ so that the line is “}else{“, otherwise the script fails and crashes.

*DB: This seems to be a machine-specific issue as I could not reproduce this error (crashing script) but I removed the line break.*

**File: 08TarlingEtAlSummerGrowth.R**

- Line 43 & 47: “TarlingEtAl2006IMPRounded” object is not defined in the script, so could not run the rest of the code

*DB: I forgot to update the function calls in lines 43 and 47 after renaming the function from TarlingEtAl2006IMPRounded to TarlingEtAl2006IMP (the option of rounding the IMPs is now passed to the function as an argument). This is now fixed and the script should work as it is.*

**File: 10ManuscriptPlots.R**

- Code for p3 (lines 87-118): The line for the average trajectory for the Fach et al 2002 model does not match the figure in the manuscript (see below). Please double check the code in File 04FachEtAlSummerGrowth.R

*DB: Thank you for spotting this issue. The problem was that the chlorophyll a dataset was getting flipped upside down during the simulations in line 110, which led to wrong results. This bug is now corrected, and the code now fully reproduces the trajectories presented in the manuscript.*

- Line 155: object “modelResultsSub” not defined so modelResultsCV cannot be calculated

*DB: The object modelResultsSub is now defined in the previous line in an updated version of the script*

- Lines 181-202: A section of the maps appears in the modelDiff plots which appears as “NA” in Fig 5 (see yellow circled region in figure below of the differences from the Atkinson et al 2006 model)

*DB: Upon closer inspection, I realized that the issue is not the presence of the low growth rates in the region marked with the yellow ellipse but that the Southern Polar Front Mask used to mask the results is slightly rotated in the Figure that was produced from the code we provided in the github repository (see original plot from Figure 5 on the right side for comparison, the SFP-mask has the same shape but it is slightly rotated clockwise). The reason is that in the original code we published on github, the SFP-mask had a different coordinate reference system than the model results which led to the observed differences. The code is now corrected and the SFP mask is transformed into the same CRS as the data prior to masking, which results in the right map, which is identical to those shown in Figure 5.*

- Line 357: “library(geomtextpath)” needs to be run before p1 can be generated

*DB: Thank you, library(geomtextpath) is now added at the top of the script*

- Line 429: change to “ggsave('plots/RyabovChla.pdf', plot = p1, width = 7, height = 6)” so that the file path is relative to the working directory (same applies to line 450, 473, 494, 514)

*DB: We now changed the file paths from absolute to relative paths*

**Other changes**

**S1 Fig.:** *The growth rates of the model of Atkinson et al. (2006) in the old version were erroneously calculated using the “juvenile” parameterization instead of the “all krill” parameterization as would have been appropriate for a 50 mm individual. We corrected this accordingly.*

**Introduction of the model of Tarling et al. (2006):** *We now state more explicitly that the model we refer to as Tarling et al. (2006) is a composite model that is structurally analogous to the model of Wiedenmann et al. (2008), but has not been used before exactly as we do in this study. The main reason for including it was its very close relationship to the (comparatively) frequently used growth models of Atkinson et al. (2006), and to explore the sensitivity of the predictions of models incorporating moulting as a process of growth and development to the choice of the IMP function of Kawaguchi et al. (2006) or Tarling et al. (2006)*

*We are convinced that this is a very important model to include, especially since moulting was highlighted as an important factor of krill development (e.g. in Constable and Kawaguchi 2018). Nevertheless, we thought it would be important to point out this detail.*
